# Bioinspired, Guanidinium, and Indole Modified Poly(glycidyl ether)s as Highly Efficient Vectors for Polyplex‐Mediated Gene Delivery

**DOI:** 10.1002/marc.202500873

**Published:** 2026-01-18

**Authors:** Markus Kötzsche, Andreas Dzierza, Lennert Sölter, Jan Egger, Kjell Cornelis, Andreas Stihl, Felix H. Schacher, Dagmar Fischer, Kalina Peneva

**Affiliations:** ^1^ Friedrich Schiller University Jena Institute of Organic and Macromolecular Chemistry (IOMC) Jena Germany; ^2^ Friedrich‐Alexander‐Universität Erlangen‐Nürnberg Division of Pharmaceutical Technology and Biopharmacy Erlangen Germany; ^3^ Jena Center for Soft Matter Jena Germany; ^4^ FAU NeW ‐ Research Center New Bioactive Compounds Erlangen Germany

**Keywords:** gene delivery, guanidinium, hydrophilic‐hydrophobic balance, indole, polyplexes

## Abstract

Allyl glycidyl ether and 2‐methoxyethyl glycidyl ether were copolymerized via anionic ring‐opening polymerization and subsequently functionalized with guanidinium and indole groups through a post‐polymerization thiol‐ene reaction. This modular approach yielded eight polymers with systematically varied hydrophilic‐hydrophobic balance, carrying 50–92 mol% guanidinium and 0–22 mol% indole. The polymers featured molar masses between 10.1 and 15.7 kg/mol with a dispersity of around 1.3. Polyplexes were formulated using plasmid DNA and characterized with respect to their physicochemical properties including DNA binding affinity, surface charge, and particle size as well as their transfection efficiencies and polymer in vitro cytotoxicity. All polymers were able to form stable complexes and protected their cargo against enzymatic degradation. An additional hydrophilic monomer did not influence physicochemical characteristics, but increased polymer cytotoxicity. Transfection studies in CHO‐K1 cells revealed a strong dependence on polymer hydrophobicity: polymers with medium indole content outperformed both more hydrophilic and more hydrophobic analogues, reaching efficiencies above the gold standard poly(ethylene imine). These results underline the critical role of balancing hydrophilic and hydrophobic groups in side‐chain functionalized poly(glycidyl ether)s for safe and effective gene delivery.

## Graphical abstract

Guanidinium‐ and indole‐functionalized poly(glycidyl ether)s were developed as non‐viral gene delivery vectors with tunable hydrophilic–hydrophobic balance. All polymers formed stable DNA polyplexes, while moderate indole content yielded the highest transfection efficiency at low N/P ratios. The study highlights hydrophobic balance as a key design principle for efficient and safe polymer‐based gene delivery.

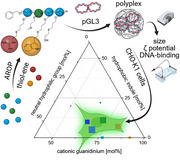

## 1 Introduction

Gene delivery provides new opportunities to treat “undruggable” diseases that cannot be addressed with conventional small‐molecule therapies [1]. However, efficient intracellular delivery of negatively charged nucleic acids remains challenging. After efficient packaging, nucleic acids must be protected from enzymatic degradation, transverse the negatively charged cell membrane, escape from endosomes, and ultimately release their cargo in the cytoplasm [2]. In addition to viral vectors limited by safety concerns and immunogenicity, non‐viral vectors have emerged as attractive alternatives to address these challenges [3]. Positively charged or protonable polymers can complex DNA or RNA via electrostatic interactions, forming polyplexes [4]. Among these poly(ethylene imine) (PEI) is considered the gold standard in transfection studies with polyplexes, and has been extensively modified in terms of molar mass, degree of branching, and chemical modifications to improve its efficiency [5, 6].

In both PEI and poly(amidoamine)s (PAMAMs), another widely studied class, such modifications often involve changes in the type and distribution of protonable groups, typically shifting from secondary to tertiary or quaternary amines. In many cases, net charge is reduced due to the conversion to amides or carbamates, since the amine groups themselves serve as reactive sites within the polymer [7, 8, 9, 10, 11]. One strategy to overcome this limitation is to employ polymer backbones bearing chargeable groups in the side chains, rather than in the main polymer chain. This approach enables the incorporation of various functional groups while preserving the polymer's cationic character.

Backbones such as poly(methacrylates) [12], polyphosphoesters [13, 14], and dextranes [15], known for their biocompatibility, have been explored to minimize cytotoxicity. Placing chargeable groups in the polymer side chains also allows to use groups with delocalized charge as, inspired by arginine‐rich cell‐penetrating peptides, guanidinium groups, which were reported for use in poly(methacrylates) and compared with amines regarding DNA binding efficiencies related to their arrangement as well as their respective amount in the polymer before [12]. Guanidinium groups offer a wide range of benefits compared to their amine counterparts due to its high basicity and thus to a higher charge ratio at physiological pH, which further is delocalized in a planar structure, offering a variety of interactions with hydrophobic aromatic structures as well as with hydrophilic negative charges from the DNA as well as from the target cells [16, 17]. Beyond electrostatics, hydrophobic effects are also crucial during transfection and cargo release, Sabin et al., highlighted the importance of hydrophobicity in improving polymer–membrane interactions [18].

Following this rationale, strategies to enhance PEI efficiency often involve the grafting of hydrophobic groups as shown for alkyl chains or cholesterol‐modified PAMAMs. In both systems, moderate hydrophobicity yielded optimal transfection efficiency, whereas excessive hydrophobicity led to reduced performance [8, 11].

For polymers with the charged group in the side chains, incorporating the lipophilic group either as linker between backbone and charge [19] or as separate monomer is possible. Nelson et al., synthesized poly[(ethylene glycol)‐block‐[(2‐(dimethylamino)ethyl methacrylate)co‐(butyl methacrylate)] diblock copolymers, allowing to regulate polymer hydrophobicity, directly linked to transfection efficiency, following the trends as described above for PEI [20]. Furthermore, this study is also an example for influencing the behavior of charged polymers by a PEG block [21, 22, 23, 24] to modulate protein adsorption [25], protection against enzymatic degradation [26], to improve blood circulation, and provide stealth properties [27, 28, 29, 30, 31].

The use of glycidyl ether in anionic ring‐opening polymerization (AROP) gives facile access to side‐chain functionalized polyethers [32, 33]. The most accessible groups are alkyl chains [34, 35, 36, 37, 38, 39], while poly(allyl glycidyl ether) (P(AGE)) that can be used as electrolyte itself [40], offers a platform for post‐polymerization functionalization for example by thiol‐ene reactions or metathesis [41, 42, 43, 44, 45, 46]. This allows the introduction of groups like benzimidazole [47], trifluoromethanesulfonamide [48], carboxylic acids [49], or glucose [50], which otherwise would require protection groups [51]. Although P(AGE) has a similar structure as PEG, its mixing properties are effected by the side‐chain [52]. Similar glycidyl ethers can be based on bio‐derivate acyclic terpenes [53].

Amine‐grafted P(AGE) [54, 55], alkyl‐amine containing glycidyl ethers [56], and amines derived from epicyanohydrin [57, 58] were applied for cryoprotection [59], as antimicrobial polymers [60], for stimuli‐responsive polymers [61, 62, 63, 64], as battery electrolytes [65], and for complex coacervation [66]. In comparison to the amine group, the cationic group of guanidinium‐grafted P(AGE) provides a charge in a larger pH ideal for complex coacervation [67, 68, 69, 70, 71, 72, 73].

The potential of both guanidinium‐ and amine‐functionalized glycidyl ethers was further explored for nucleic acid delivery, underscoring the superior nucleic acid interactions of the guanidinium group compared to its amine counterpart [73, 74, 75, 76]. Following the principles outlined for PEI, combining cationic groups with hydrophobic moieties such as tyrosine or phenylalanine has further improved transfection efficiency [77]. In this study, we build on this rationale and describe the synthesis, characterization, and application of poly(glycidyl ether) polymers functionalized with guanidinium groups together with indole moieties. Indole has previously been identified as the most favorable hydrophobic side chain among various aromatic groups in poly(methacrylamides), owing to its ability to engage in *π*–*π* interactions and amphiphilic contacts [78, 79, 80]. Here, we investigate whether tuning the hydrophilic–hydrophobic balance by combining guanidinium and indole groups in poly(glycidyl ethers) can yield efficient and biocompatible gene delivery vectors.

## 2 Experimental

### 2.1 Poly(glycidyl ether) Copolymers

The polymerization was conducted inside a glove box. For the initiator solution, 249 mg (2.00 mmol, 1 eq) naphthalene were dissolved in 4.3 g (4.8 mL) dry tetrahydrofuran (THF) and 76 mg (1.95 mmol, 1 eq) potassium were added to obtain a dark green solution, which was then added to 41 mg (0.379 mmol) benzyl alcohol until the mixture maintained green. The monomers, allyl glycidyl ether (AGE) and 2‐methoxyethyl glycidyl ether (MEGE) were filled in a tube (Table S1). Afterward, the initiator solution was added and the mixture was stirred for 22 h at room temperature. The polymerisation was terminated with 1 mL methanol. The crude polymer was purified by dialysis against methanol with a 1 kD MWCO membrane. The solvent was removed under vacuum.

### 2.2 Grafted Poly(glycidyl ether)s

The unfunctionalized polymer was dissolved in dry dimethylformamide (DMF) and added to (2‐mercaptoethyl)guanidine, 2‐(1H‐indol‐3‐yl)ethane‐1‐thiol, tris(2‐carboxyethyl)phosphine hydrochloride, and 2,2‐dimethoxy‐2‐phenylacetophenone in a tube. The mixture was degassed with argon for 30 min and then irradiated with UV light (365 nm) at room temperature for 2 h. The polymers were purified with dialysis against methanol/MilliQ water using a 3.5 kDa MWCO membrane and obtained by freeze‐drying.

Due to incomplete grafting, 100 mg functionalised polymer, 200 µL 2‐mercaptoethanol, and 30 mg 2,2‐dimethoxy‐2‐phenylacetophenone were filled in a tube and dissolved in 1 mL DMF/water 1/1 mixture. The mixture was degassed with argon for 30 min and then irradiated with UV light at room temperature for 1 h. The polymers were purified with dialysis against MilliQ water using a 3.5 kDa MWCO membrane and obtained by freeze‐drying.

### 2.3 Plasmid Preparation

Plasmid DNA (pDNA) for transfection experiments was obtained via transforming pGL3 control plasmid DNA (Promega GmbH, Walldorf, Germany), encoding for firefly luciferase, in E. coli K12 TG1 (DSM 6056, DSMZ ‐ German Collection of Microorganisms and Cell Cultures GmbH, Braunschweig, Germany). Frozen bacterial culture was thawed on ice and the pellet containing the bacteria was resuspended in sterile ice‐cold CaCl_2_ solution (100 mm, Carl Roth, Karlsruhe, Germany) after centrifugation. Plasmid DNA was transformed into the bacteria via heat shock at 42°C. Bacterial suspensions were transferred to LB agar plates containing ampicillin (LB medium, agar, and ampicillin from Carl Roth) to allow selection of transformed bacteria. After growing overnight, single colonies of the plates were picked and transferred to liquid culture (LB medium with 100 µg mL^−1^ ampicillin) and incubated at 37°C under shaking. E.Z.N.A Plasmid Maxi Kit (Omega Bio‐tek, Norcross, USA) was used according to the manufacturer's protocol to isolate and purify plasmid DNA. Isolated pDNA was analyzed via gel electrophoresis as described in gel electrophoresis section to test the topology of the obtained DNA. To determine DNA concentration, the NanoQuant Plate of the Tecan Spark Control (Tecan Group Ltd., Männedorf, Switzerland) was used, applying the automated NanoQuant Nucleic Acid Quantitation method, using DNA elution buffer of the plasmid purification kit as blank.

### 2.4 Polyplex Formulation

Polyplexes were formulated in a buffer containing 10 mm HEPES ((4‐(2‐hydroxyethyl)‐1‐piperazineethanesulfonic acid; Carl Roth) and 5% glucose (HBG; Sigma Aldrich, Darmstadt, Germany) at neutral pH. To characterize physicochemical characteristics of polyplexes, herring testes DNA (DNA) (Sigma Aldrich) was used as model nucleic acid, whereas for gel electrophoresis and biological efficiency, pDNA (pGL3 control vector for biological characterization; pBR322 pDNA from CarlRoth with comparable number of base pairs for binding experiments) was used. For all experiments, concentration of DNA was maintained constant at 20 µg mL^−1^ in polyplex dispersions. Polymers and linear poly(ethylene imine) (LPEI, 2500 g mol^−1^, Polysciences Inc., Hirschberg an der Bergstraße, Germany) were dissolved to 5 mg mL^−1^ and in order to form polyplexes with different N/P ratios (ratio of number of basic nitrogen groups (N) in the polymer to negative phosphate anions (P) in the DNA backbone), polymer solutions were diluted in HBG to the appropriate concentrations needed for the specific N/P ratio in a volume corresponding to half of the final volume of the polyplex dispersion. The fixed amount of DNA was prepared in the remaining half of the volume and in order to formulate polyplexes, polymer solutions were pipetted to the DNA solution, vortexed immediately for 10 s and allowing to form for 10 min before use in experiments [81].

### 2.5 Quantification of DNA Binding

The ability of the polymers to complex DNA was investigated using AccuBlue High Sensitivity dsDNA Quantitation Kit (Biotium, Fremont, USA; obtained via VWR, Darmstadt, Germany). Samples of polyplex dispersions (1 µg herring testes DNA per 50 µL, N/P 2–20) were pipetted on black 96‐well plates in triplicates, containing 100 ng DNA each. Following the manufacturer's protocol, 200 µL working solution (mixture of 100X enhancer and quantification buffer in ratio 1:100) were added per well and fluorescence was measured in the Tecan Spark Control plate reader (λ_Ex_ = 485 nm, λ_Em_ = 530 nm) after a 10 min incubation step at room temperature in the dark at 200 rpm. Values of non‐complexed DNA were calculated as percentage of the fluorescence signal of free DNA solution, set as 100%, after subtracting the signal of HBG serving as blank. Polymer solutions in concentrations as in NP 20 without DNA were used to exclude interference of the polymers with the used assay. Results were calculated as mean and standard deviations of three independent repetitions (*n* = 3).

### 2.6 Horizontal Agarose Gel Electrophoresis

In order to detect uncomplexed DNA, in addition to the AccuBlue assay performed with herring testes DNA, agarose gel electrophoresis was carried out with pDNA. Polyplex dispersions containing 1 µg pDNA in 50 µL were mixed with 12 µL of loading buffer ((40 mm tris(hydroxymethyl) aminomethane (TRIS; Carl Roth), 1 mm ethylenediaminetetraacetic acid disodium salt (EDTA; Sigma Aldrich), 50% (v/v) glycerol 85% (Carl Roth)). Samples of 12 µL were then loaded onto a 1% agarose (peqGold Universal Agarose, VWR) gel containing 5 µL of the 1:1000 GelRed solution (Biotium) as intercalating dye for DNA fluorescence detection [82]. Free pDNA solution and polymer solution without DNA in concentration as in N/P 20 were used as controls, while aqueous bromophenol blue solution (0.5%; m/V, Carl Roth) mixed with loading buffer (mixing ratio 1:1) served as separate dye front marker. Electrophoretic separation was performed in TAE running buffer (40 mm TRIS, 1 mm EDTA, and 0.1% acetic acid (Sigma Aldrich)) for 1 h at 80 V (BioRad PowerPac 1000, Bio‐Rad Laboratories GmbH, Feldkirchen) in a MidiPlus gel chamber (VWR). The gel was placed on a UV transilluminator (λ_Ex_ = 312 nm, Intas GmbH, Goettingen, Germany) and images were captured using DocPrint CX3 gel documentation system (Vilber Lourmat, Collegien, France), following image processing with BioVision software (Vilber Lourmat).

### 2.7 Dynamic Light Scattering Techniques

Dynamic light scattering was used to determine hydrodynamic diameter (HD) and polydispersity index (PDI) of respective polyplexes and laser Doppler anemometry was used to investigate the zeta potential (ZP) of the particles, both using the Zetasizer Ultra (Malvern Panalytical, Kassel, Germany). Polyplexes were prepared with herring testes DNA at different N/P ratios (2, 5, 10, and 20) in HBG as described above and were transferred in the high concentration zeta cell (ZEN1010, Malvern Panalytical) via the Luer Lock connection. Settings were adjusted to a viscosity of 1.02 mPa·s and a refractive index of 1.34 as characteristics of HBG and measurements were run at 25°C three times per sample with the default backscattering angle of 174.8° for HD and 12.69° for ZP evaluations (laser wavelength: 633 nm; 10 mW HeNe laser). Collected data was analyzed using ZS Xplorer software (v1.3.1.7, Malvern Panalytical, calculated Z‐average (default Cumulants Fit) was used for HD interpretation). Measurements were repeated independently twice (*n* = 3).

### 2.8 Test on Protection Against Degradation

To study the ability of polyplexes to protect DNA as cargo from degradation, polyplexes were prepared in the same manner as described above with 1 µg pDNA in 50 µL HBG. After the above described 10‐min incubation for polyplex assembly, 2 µL of DNase I solution (ready‐to‐use solution with 2.5 kunitzU per µL, containing 10 mm Tris‐HCl (pH 7.5), 10 mm CaCl_2_ and 10 mm MgCl_2_; Thermo Fisher Scientific, Darmstadt, Germany) was added, followed by 45 min of incubation at 37°C in a thermocycler (Biometra TOne, Analytik Jena, Jena, Germany). In the next step, the samples were cooled before retrieval, and 2.5 µL of 0.1 M EDTA solution was added to each vial. To inactivate DNase I, the samples were exposed to 70°C for 35 min in the thermocycler. After again cooling to 20°C for sample withdrawal, 10 µL of heparin solution (2.5 U µL^−1^, heparin sodium salt from pork intestine mucosa, 20 kDa, Carl Roth) were pipetted to each sample to facilitate DNA release from the polyplexes for subsequent gel electrophoresis. A final incubation at 37°C for 20 min ensured efficient DNA displacement before loading onto an agarose gel, as described in agarose gel electrophoresis section. Control samples were processed using free pDNA solutions at the same concentration as in the polyplexes. These controls included (i) untreated pDNA, (ii) pDNA that underwent the same thermocycler conditions but with adding 2 µL of HBG instead of DNase solution and (iii) pDNA processed identically to the polyplex samples.

### 2.9 In Vitro Cytotoxicity

To assess the viability of cells in presence of the polymers, the CellTiter‐Glo Luminescent Cell Viability Assay (Promega) was performed on L‐929 cells (ACC 2, DSMZ ‐ German Collection of Microorganisms and Cell Cultures GmbH), a standard cell line for toxicity testing according to the ISO 10993‐5 guideline [83]. Cells were seeded into white 96‐well cell culture plates (Thermo Fisher Scientific) at a density of 7500 cells in 200 µL per well in complete growth medium (RPMI 1640 Medium with GlutaMAX Supplement, supplemented with 10% FBS; both from Gibco, Thermo Fisher Scientific). In the next step, the cells were treated with polymer solutions at varying concentration after a 24‐h incubation at 37°C in a humidified atmosphere with 5% CO_2_. Polymer dilutions steps ranging from 500 to 1.95 µg mL^−1^ were processed in complete growth medium. After removing the supernatant of the cells, polymer dilutions were applied in quadruplicates per cell culture plate by adding 100 µL per well. Cell viability was measured after an additional 24‐h incubation step at the previous mentioned conditions, and readout was performed following the manufacturer's instructions by adding 100 µL of the assay's working solution. Cell culture plates were incubated at 450 rpm for 2 min at room temperature, followed by further 10 min incubation without shaking at the same temperature. The Tecan Spark Control was used to measure luminescence per well.

Cells cultured in full growth medium without polymer treatment were set as 100% viable, while full growth medium with thiomersal solution (final concentration 0.02%, Carl Roth) was used as control with minimal metabolic activity [84] and full growth medium containing no cells served as blank. Relative viability was calculated as percentage of the 100% viable control, after subtracting the blank signal. Experiments were run three times independently for each polymer (*n* = 3). Origin 2021 was used for curve fittings and IC_50_ calculations using the logistic function.

### 2.10 In Vitro Transfection Efficiency

For transfection experiments, 50 000 CHO‐K1 cells (ACC 110, DSMZ ‐ German Collection of Microorganisms and Cell Cultures GmbH) were seeded per well in 12‐well cell culture plates (Greiner Bio‐One, Kremsmünster, Austria). After allowing to grow for 24 h while incubating at 37°C with 5% CO_2_ in a humidified atmosphere, 200 µL of polyplex samples (N/P 3, 5, 10, and 20) containing 4 µg pGL3 pDNA were prepared as described in polyplex formulation section in buffer (150 mm NaCl, 10 mm HEPES, pH 7.4). As positive control, polyplexes formulated with LPEI, known for its high transfection efficiency [15, 24, 78, 79, 85], was used at an N/P ratio of 10. As blank, buffer alone was used and naked pDNA was added to exclude unspecific uptake. After the described incubation of 10 min, polyplex dispersions were added to cell culture wells containing 2 mL fresh culture medium (F12 Nut Mix (Ham) supplemented with 10% fetal bovine serum (FBS), both from Gibco, Thermo Fisher Scientific). After 4 h of incubation, the medium was aspirated, and cells were washed with DPBS (Dulbecco's Balanced Salt Solution, Gibco, Thermo Fisher Scientific), before adding 2 mL of fresh medium per well. The cells were then incubated for an additional 44 h. Transfection efficiency was investigated using the Luciferase Assay System (Promega) after inspecting the cell culture by light microscopy (Inverted microscope AE2000; Motic, Barcelona, Spain). Shortly, cells were washed with DPBS after aspirating the medium and lysed with 150 µL of Cell Culture Lysis Reagent (CCLR, Promega). The lysate was transferred to reaction tubes (Carl Roth) and centrifuged at 12 000 g and 4°C for 2 min (Allegra 64R, Beckmann Coulter, Krefeld, Germany) according to the manufacturer's instructions. 20 µL of each supernatant were pipetted in duplicates into a white 96‐well plate. Luminescence was measured using the Tecan Spark Control using its autoinjector, injecting 100 µL of the assay's working solution and recording the signal immediately over 10 seconds per well. The protein content per sample was determined using the bicinchoninic acid assay (Pierce BCA Protein Assay Kit, Thermo Fisher Scientific). Of each supernatant, 25 µL were transferred in duplicates to a transparent 96‐well plate (Greiner Bio‐One). To remove reducing agents from CCLR in the lysates, probably interfering with the assay, 10 µL of 9.25 mg mL^−1^ iodoacetamide solution (Thermo Fisher Scientific) was added to each well. Albumin dilutions were prepared as calibration curve and processed in the same manner as the samples, allowing calculation of total protein content per measured sample. Upon 15 min of incubation, 200 µL of the assay's working solution (50:1 dilution of BCA reagent A and BCA reagent B) were pipetted in each well, and the plate was incubated at 37°C for 45 min. Absorbance was measured at 562 nm using the Tecan Spark Control plate reader. Transfection efficiency values were given in relative light units per total amount of protein in each sample. Experiments were repeated independently twice and results were calculated as mean of the in total four independently transfected wells (*n* = 4 ± standard deviation).

## 3 Results and Discussion

### 3.1 Polymer Synthesis

**SCHEME 1:**
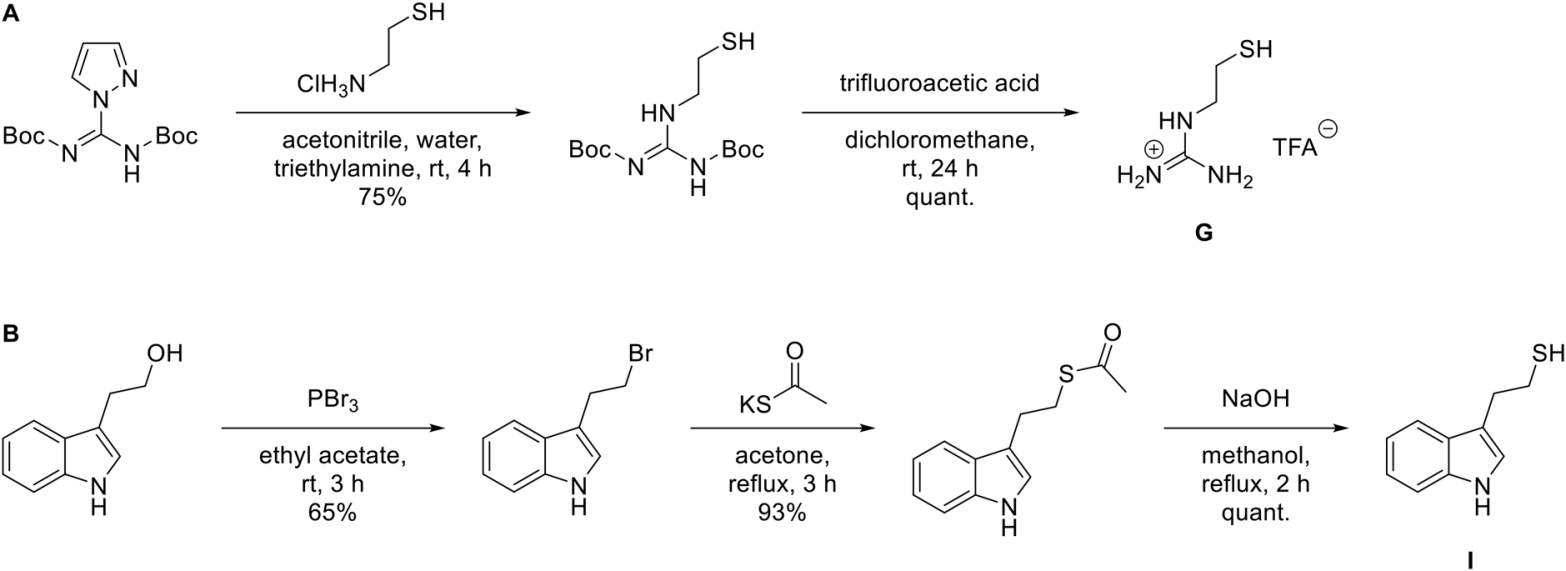
Synthetic routes for the grafting agents: (A) synthesis of (2‐mercaptoethyl)guanidine (G); (B) synthesis of 2‐(1H‐indol‐3‐yl)ethane‐1‐thiol (I) from 3‐indoleethanol via bromination, thioacetate substitution, and hydrolysis.

Guanidinium‐ and indole‐functionalized poly(glycidyl ether)s were prepared by post‐polymerization thiol‐ene reaction of poly(allyl glycidyl ether). The guanidinium grafting agent (2‐mercaptoethyl)guanidine (G) was synthesized in one step with a yield of 75% and deprotected prior to use (Scheme 1 and Figure S6). The indole‐containing grafting agent 2‐(1H‐indol‐3‐yl)ethane‐1‐thiol (I) was synthesized from 3‐indoleethanol in three steps. First, the alcohol was converted to a bromine in 65% yield using phosphorus tribromide, which was then substituted by thioacetate in 93% yield. The free thiol was obtained by hydrolysis of the ester under basic conditions (Figures S2–S4).

**SCHEME 2:**
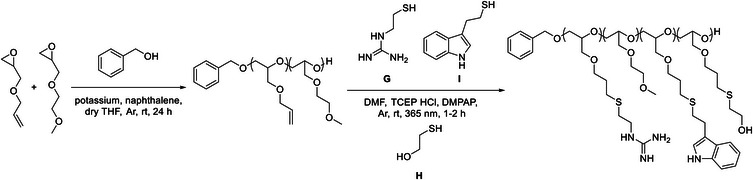
Preparation of functional poly(glycidyl ether)s: statistical copolymerization of allyl glycidyl ether with 2‐methoxyethyl glycidyl ether by anionic ring‐opening polymerization, followed by post‐polymerization thiol‐ene grafting with guanidinium and indole groups.

2‐Methoxyethyl glycidyl ether (M) was prepared in one step from epichlorohydrin and methoxyethanol in 84% yield (Figure S1). A homopolymer of allyl glycidyl ether and a statistical copolymer with 22 mol% 2‐methoxyethyl glycidyl ether as hydrophilic group were prepared via anionic ring‐opening polymerization (Scheme 2 and Figures S7 and S8). A potassium‐naphthalene‐benzyl alcohol mixture was used as initiator solution and the polymers were purified by dialysis against methanol. The molar mass distributions were characterized with size‐exclusion chromatography in THF and poly(ethylene glycol) calibration (Figure S9). Both polymers exhibited a shoulder at twice the peak maximum resulting in dispersities of 1.22 for the homopolymer and 1.36 for the copolymer. The number average molar masses were 7.9 kg mol^−1^ for the homopolymer and 9.3 kg mol^−1^ for the copolymer.

Each polymer was then grafted with (2‐mercaptoethyl)guanidine and 0, 5, 10, or 20 mol% 2‐(1H‐indol‐3‐yl)ethane‐1‐thiol in DMF using 2,2‐dimethoxy‐2‐phenylacetophenone as photoinitiator. Tris(2‐carboxyethyl)phosphine hydrochloride was added to maintain the presence of free thiols. After purification via dialysis in methanol/water, small amounts (1–17 mol%) of allyl groups were still visible in the ^1^H‐NMR spectra (Figures S10–S17). Complete functionalization was achieved with an excess of 2‐mercaptoethanol (H) in a second grafting step (Figures S18–S25). Due to overlapping signals with the other grafted side‐chains, the first NMR spectra were used to calculate the compositions.

The polymers without the MEGE comonomer retained more allyl groups after the first grafting yet exhibited higher guanidinium content overall (63–92 mol%, Table 1). Indole incorporation ranged from 0 to 20 mol%. As predicted from previous studies, polymers with intermediate hydrophobicity are expected to show optimal transfection [8, 80]. Molar masses increased after grafting, while dispersities remained low (Figure S26).

**TABLE 1:** Composition and molar mass of guanidinium‐ and indole‐functionalized poly(glycidyl ether)s. Polymers were obtained from allyl glycidyl ether (AGE) or AGE/2‐methoxyethyl glycidyl ether (MEGE) copolymers, followed by thiol‐ene grafting with (2‐mercaptoethyl)guanidine and 2‐(1H‐indol‐3‐yl)ethane‐1‐thiol. Shown are the molar fractions of guanidinium (G), MEGE (M), indole (I), and residual hydroxyethyl (H) groups, together with the number‐average molar mass (M_n_) and dispersity (Đ).

Polymer	Guanidinium [mol%]	2‐Methoxyethyl [mol%]	Indole [mol%]	2‐Hydroxyethyl [mol%]	M_n_ [kg mol^−1^]	Đ
**P(G_92_H_8_)**	92	−	−	8	10.1	1.18
**P(G_82_I_7_H_11_)**	82	−	7	11	12.0	1.23
**P(G_72_I_11_H_17_)**	72	−	11	17	13.5	1.30
**P(G_65_I_22_H_13_)**	65	−	22	13	13.5	1.30
**P(G_77_M_22_H_1_)**	77	22	−	1	14.5	1.28
**P(G_69_M_22_I_5_H_4_)**	69	22	5	4	15.7	1.39
**P(G_64_M_22_I_10_H_4_)**	64	22	10	4	15.1	1.39
**P(G_50_M_22_I_20_H_8_)**	50	22	20	8	15.2	1.42

### 3.2 DNA Binding Efficiency

**FIGURE 1:**
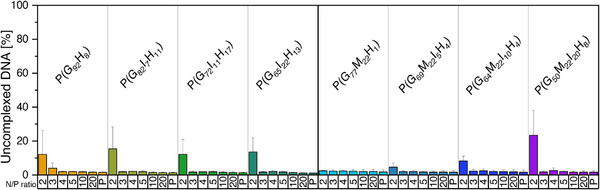
Quantification of uncomplexed DNA at different N/P ratios (2–20), using the AccuBlue High Sensitivity dsDNA Quantitation Kit. Free polymers as in N/P 20 without DNA (P) was used as control for each polymer to exclude polymer signals. Experiments were performed in triplicates and run three times independently (*n* = 3; mean ± SD).

Quantification of non‐complexed DNA was conducted using AccuBlue High Sensitivity dsDNA Quantitation Kit as fluorescent dye exclusion assay, with polyplexes with N/P ratios ranging from 2 to 3–5, and 10–20. At an N/P ratio of 2, some moderate signals from uncomplexed DNA were still detectable, which are completely diminished when rising the N/P ratio to and above 3, revealing signals in a range of the polymer alone without DNA (Figure 1), indicating a high binding efficiency of the tested polymers, independently from the exact polymer composition.

These data are in good accordance with the performed gel electrophoresis with pDNA, where no detectable signals outside the gel pockets were visible after electrophoretic separation, indicating full complexation of the pDNA and thus hindering of the movement of pDNA in the gel driven by the applied voltage (Figure S27) [86]. Polymers alone as control gave no signals and naked pDNA appeared with two bands for the circular and supercoiled form.

Importantly, neither the indole moiety nor the MEGE comonomer impaired DNA complexation. Thus, guanidinium residue dominated the complexation process, consistent with their high charge density and planar delocalization, enabling stable interactions with DNA phosphates. This behavior differs from amine‐based polymers, where the protonation state and charge density are more sensitive to chemical modifications, and side‐chain substitutions can therefore strongly influence DNA binding [77].

### 3.3 Protection from Degradation

One major concern in the field of nucleic acids delivery is not only the binding of the cargo, but also the protection from degradation by physical or electrostatic barriers to allow the delivery of intact DNA to the cell. To qualitatively study the ability of the eight tested polymers to protect their cargo in dependency of polymer composition and N/P ratio, polyplexes were incubated with DNase I and after enzyme inactivation, and pDNA was released from the polymers through electrostatic displacement with heparin. Gel electrophoresis with GelRed as intercalating fluorescent dye was applied afterward, allowing a qualitative insight on the pDNA integrity (Figure S28). Free pDNA revealed two characteristic bands after staining representing the open circular and the supercoiled conformation that was not changed for pDNA undergoing the same treatment as the polyplexes, but without DNase. No patterns were visible for free pDNA exposed to the enzyme, implying full degradation in absence of protection.

For all polymers, the supercoiled form of the plasmid could be partially detected at all N/P ratios indicating protection from cleavage through DNase. Additionally, for P(G_77_M_22_H_1_) additional parts of the intact open circular form were visible. For all polymers only a partial pDNA release from the polyplexes could be observed as shown by a high fluorescence in the loading pockets at the origin which is related to the high binding affinity of the polymers and favors stabilization of the pDNA [85]. The signal intensities of the released pDNA differ slightly between the gels, which might be suggested not to be due to partial degradation but rather to differences in pDNA release from the polyplexes. Conclusively, these data suggest the ability of the copolymers not only to bind pDNA, but also to complex it compact enough to avoid pDNA depletion, fulfilling one major requirement for their potential suitability for gene delivery [87, 88].

### 3.4 Physicochemical Polyplex Characteristics

**FIGURE 2:**
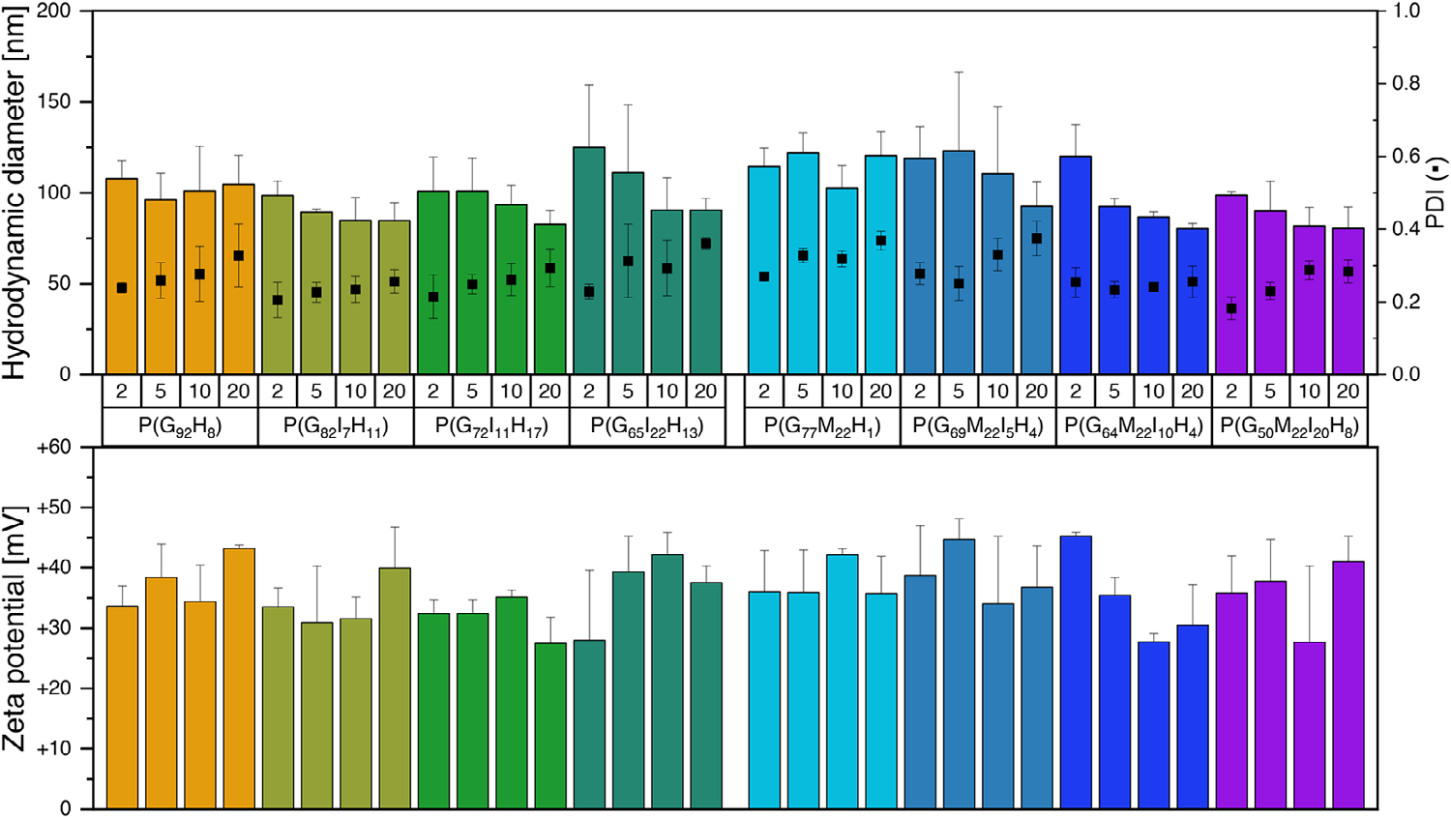
Hydrodynamic diameters, polydispersity indices (PDI) and zeta potentials of polyplexes (N/P ratio 2–20) of different polymers, evaluated via dynamic light scattering and laser Doppler anemometry, respectively. Measurements were run three times per sample and repeated independently twice (*n* = 3, mean ± SD).

Dynamic light scattering (DLS) was used to investigate hydrodynamic diameters (HD) and polydispersity indices (PDI) of formed polyplexes. For all copolymers, HDs were lower than 200 nm with values ranging from 80 to 125 nm (Figure 2). In the first polymer group with the more hydrophobic polymers all hydrodynamic diameters were almost comparable between N/P ratio 2–20 taking the standard deviations into consideration. Within the second polymer group carrying an additional hydrophilic side chain, a slight tendency of lower mean hydrodynamic diameters for the two more hydrophobic polymers P(G_50_M_22_I_20_H_8_) and P(G_64_M_22_I_10_H_4_) could be suggested. This could probably be related to disturbing effects of the additional hydrophilic group on DNA binding, as described for example for copolymers e.g., with sugar chains in the polymer [8, 89]. Furthermore, this effect is counterbalanced through adding hydrophobic indole moieties, revealing higher hydrophobicity at a level comparable to the other polymers. This data‐derived trend was statistically significant e.g., at N/P 20, where P(G_77_M_22_H_1_), as the most hydrophilic polymer in this methoxy‐containing polymer group, showed a significant difference of particle size compared with P(G_50_M_22_I_20_H_8_) and P(G_64_M_22_I_10_H_4_) (*p* < 0.01, respectively; Tukey‐test, ANOVA performed in Origin 2021; α = 0.05) (Figure 2).

Size distribution, represented by the PDI of the polyplex dispersions, exhibited higher PDI values with rising N/P ratios, which can be explained with free or only weakly bound copolymer chains on the polyplex surface at higher N/P ratios, leading to inhomogeneous scattering signals and thus a broader distribution in DLS measurements [90].

For the lowest tested N/P ratios of 2, PDI values were smaller than 0.3 for all copolymers, indicating a moderate size distribution for polyplexes [91, 92, 93]. Interestingly, at N/P 10, the highest PDI values again were measured for the two copolymers also revealing the highest HD, further conforming the above‐mentioned hypothesis. To assess the surface charge of the aggregates, the zeta potential was measured via laser Doppler anemometry. All polyplexes revealed positive surface charges, favorable for interaction with cellular membranes and cellular uptake [94, 95] above +27 mV, but also important for stability of the dispersion, as higher surface charges lead to electrostatic repulsion of the particles [96]. Together with the HDs below 200 nm mentioned above, the particle characteristics give evidence for a suitable system for cellular uptake and thus biological activity [97, 98, 99].

### 3.5 In Vitro Cytotoxicity

**FIGURE 3:**
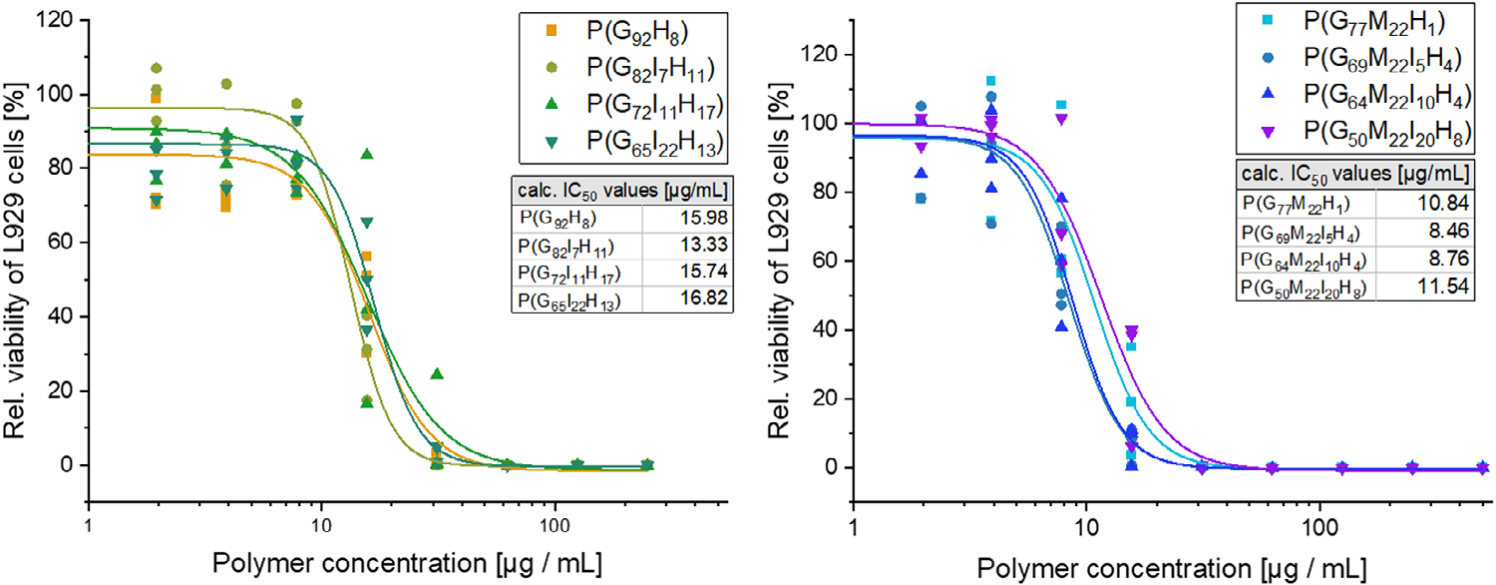
In vitro cytotoxicity of used polymers dependent on the concentration, determined with CellTiter‐Glo Luminescent Cell Viability Assay on L929 mouse fibroblasts after 24 h. Experiments were performed in quadruplicates and run three times independently (*n* = 3; dots represent mean of one independent repetition).

To evaluate the effect of the copolymers on cell viability, cytotoxicity was tested in L929 mouse fibroblast cells, using the luminescence based CellTiter‐Glo Luminescent Cell Viability Assay, which allows to determine the total amount of ATP inside one cell culture well, and correlating with the number of viable, metabolic active cells [100], using thiomersal as toxic positive control depleting metabolic activity of the cells to approximately zero percent (data not shown). Across both polymer groups with and without the methoxy side chain, cytotoxicity patterns were relatively comparable. The standard errors of the IC_50_ values ranged between 0.43 and 1.78 µg mL^−1^ (Table S3). Regarding this, within each respective polymer group (with or without methoxy groups), the IC_50_ values are quite comparable. However, the calculated mean IC_50_ values were all slightly lower for the methoxy‐containing polymers, ranging from 8.5 to 11.5 µg mL^−1^, compared to 13.3–16.8 µg/mL for the polymer series without this substituent (Figure 3). For comparison, the cytotoxicity for the gold standard linear poly(ethylene imine) (LPEI) with 2500 g mol^−1^ tested in the same setting was reported to be slightly higher (IC_50 LPEI_ of 21.1 µg mL^−1^) [101].

Although the differences between the two polymer series are not high, it can be in line with Lin et al., who reported that increased spacing between charged groups in cationic polymers correlates with higher cytotoxicity, suggested that this effect could also apply here due to the presence of the spacer monomer [102]. Second, the methoxy‐containing polymers exhibited somewhat higher molecular weights, a parameter previously reported to correlate with increased cytotoxicity in cationic polymers [103, 104]. The combination of both factors could potentially account for the slightly lower IC_50_ values observed in this group. Taken together, these results show that both polymer classes exhibit measurable but acceptable cytotoxicity in a comparable range like the linear poly(ethylene imine) standard.

### 3.6 In Vitro Transfection Efficiency

As a standard cell line for transfection experiments [105], CHO‐K1 cells were transfected with polyplexes formulated with pGL3 control plasmid DNA, encoding for luciferase, at N/P ratios 3, 5, 10, and 20. Readout of transfection efficiency was performed with the Luciferase Assay System, detecting the luminescence of luciferase expressed by transfected cells, normalized to the total protein amount per sample, measured via BCA assay.

**FIGURE 4:**
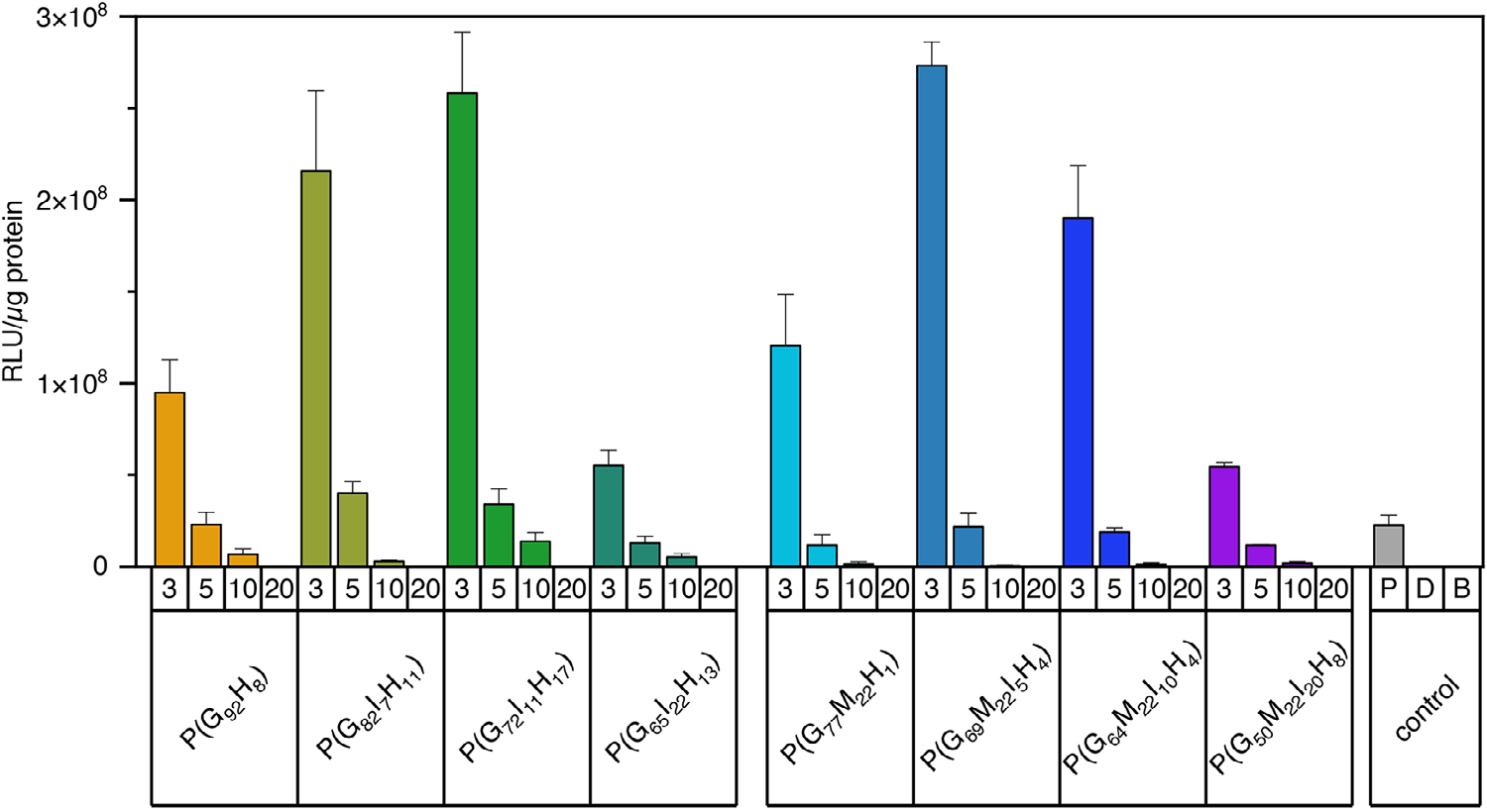
In vitro transfection efficiency of polyplexes at a variety of N/P ratios (3–20) with 4 µg pDNA in CHO‐K1 cells, expressed as relative light units (RLU) per µg protein in cell lysates, determined with Luciferase Assay System from Promega. Polyplexes with linear poly(ethylene imine) (2500 g/mol; N/P ratio 10; “P”), free plasmid DNA (“D”), and buffer only as blank (“B”) were used as controls. Experiments were conducted in two wells per run and repeated independently once (*n* = 4, mean ± SD).

First, for all polymers, the highest transfection efficiency was observed at N/P ratio of 3, while the lowest values were found at N/P ratio of 20 (Figure 4). This trend can be explained by toxicity‐related effects. Based on the above calculated IC_50_ values, polymer concentrations at N/P 10 already approached or exceeded toxic levels. For instance, P(G_92_H_8_) which contains the highest guanidinium content and therefore requires the least polymer mass to achieve a given N/P ratio, reached a concentration of 14 µg mL^−1^ at N/P 10 (close to its IC_50_ of 16 µg mL^−1^). In contrast, P(G_48_M_22_I_22_H_8_), with the lowest guanidinium content, required more polymer mass to achieve the same charge ratio, resulting in 23 µg mL^−1^ at N/P 10 – well above its IC_50_ of 11.5 µg mL^−1^. At N/P ratio 20, polymer concentrations were doubled relative to N/P 10 further exacerbating toxicity and explaining the sharp decline in efficiency at this ratio.

For cationic polymers, the charge‐associated cytotoxicity is one of the major concerns which could hamper transfection [106, 107]. The cytotoxicity of polyplexes is well‐described to increase with increasing N/P ratio because higher N/P ratios introduce excess free polymer, which is not involved in the pDNA complexation and relevant for complex stabilization, but adversely interacts with cells [108, 109]. Additionally, it has to be taken into consideration that the unbound polymer usually presents a higher toxicity than in the polyplex‐bound state [110]. In the presented transfection, a difference between polyplexes with varying N/P‐ratios was evident when cell growth and cell morphology were inspected by light microscopy as well as by BCA assay. With the increase in N/P ratio, the detectable number of cells under the light microscope immediately before cell lysis was noticeably lower (data not shown). This effect was also reflected in the amount of protein per well, measured in parallel and incorporated in the calculation of transfection efficiency. At lower N/P ratios, the protein amount was found to be higher indicating a less hampered cell growth. At N/P 3, i.e., the lowest tested ratio and thus the least toxic conditions, transfection was already highly efficient. Importantly, this was also the first ratio at which all copolymers showed complete DNA complexation as described in the DNA binding efficiency section. Taken together with the less favorable physicochemical characteristics observed at higher N/P ratios, these results demonstrate that the polymers are particularly effective gene delivery systems at low charge ratios and polymer concentrations. Notably, at N/P 3 all copolymers achieved efficiencies higher than the control polymer LPEI at N/P 10. Blank and pDNA alone resulted in nearly no luminescence.

When comparing copolymers within each group, those with the highest indole contents were consistently the least efficient. This finding agrees with our previous observations [80] and with literature reports indicating that moderate hydrophobicity maximizes transfection, whereas excessive hydrophobicity reduces performance [8, 11, 19]. In both copolymer series, introducing a small amount of indole (7 or 5 mol%, respectively) enhanced transfection roughly twofold compared to indole‐free analogues. In the series lacking MEGE, 11 mol% indole led to the highest efficiency. In contrast, for the MEGE‐containing series, increasing indole content from 5 to 10 mol% did not improve performance further, despite the expectation that additional indole would counterbalance the hydrophilicity of the MEGE side chain.

This apparent discrepancy can be explained by toxicity effects. For example, at N/P 3 P(G_64_M_22_I_10_H_4_) reached 5.5 µg mL^−1^ (concentration during transfection) close to its IC_50_ of 8.8 µg mL^−1^, whereas P(G_72_I_11_H_17_) needed only 5.1 µg mL^−1^ (concentration during transfection) and showed a higher IC_50_ of 15.7 µg mL^−1^, resulting in lower overall toxicity. Consistently, this effect can also be seen interpreting the protein amount per well separately (data not shown), with lower protein amounts for P(G_64_M_22_I_10_H_4_) compared to P(G_72_I_11_H_17_) also at lower N/P ratios, giving evidence for hindered cell growth. Considering this, for P(G_64_M_22_I_10_H_4_) its higher toxicity might counteract the probable positive effect of indole, revealing in lower transfection values due to lower cell viability and thereby decreased transcription activity.

Overall, the data from both copolymer groups confirm that medium hydrophobicity yields optimal transfection efficiencies, in line with previous findings for indole‐functionalized poly(methacrylates). This consistency underscores the transferability of the design principle: balancing hydrophilic and hydrophobic content via indole moieties can be used to maximize biological efficiency across different polymer backbones. Moreover, all polymers tested here are highly promising for nucleic acid delivery at low charge ratios, with the polymer group lacking MEGE emerging as slightly superior when balancing transfection efficiency and cytotoxicity.

## 4 Conclusions

A library of eight guanidinium‐ and indole‐grafted poly(glycidyl ether)s was successfully synthesized to systematically tune the hydrophilic–hydrophobic balance at both the polymer backbone and side‐chain levels. A homopolymer of allyl glycidyl ether and a statistical copolymer with 2‐methoxyethyl glycidyl ether were prepared by anionic ring‐opening polymerization and grafted with (2‐mercaptoethyl)guanidine and 2‐(1H‐indol‐3‐yl)ethane‐1‐thiol. The resulting polymers varied in guanidinium content (50–92 mol%), indole incorporation (0–22 mol%), and in the copolymer series, the presence of the non‐ionic hydrophilic 2‐methoxyethyl group. All copolymers were able to complex DNA efficiently and to protect it from degradation, independent of composition, confirming the dominant role of guanidinium groups in nucleic acid binding. Physicochemical characterization revealed particle sizes and distributions suitable for efficient cellular uptake, especially for lower N/P ratios. Due to this and as polymer toxicity plays an important role at higher charge ratios, all copolymers revealed as very efficient in delivering pDNA in CHO‐K1 cells already at low N/P ratios, resulting in values higher than the gold standard poly(ethylene imine). When comparing structural variations, incorporation of the additional 2‐methoxyethyl spacer only slightly increased cytotoxicity, whereas indole‐based hydrophobization did not affect cell viability. Instead, indole content strongly influenced biological activity: polymers with medium hydrophobicity (5–11 mol% indole) achieved the best transfection results, highlighting the critical importance of balancing hydrophilic and hydrophobic moieties during polymer design. Compared to our earlier work on guanidinium‐functionalized poly(methacrylamide)s [78], the poly(glycidyl ether) platform presented here enables systematic variation of side‐chain hydrophilicity and hydrophobicity through post‐polymerization modification. While both systems demonstrate the effectiveness of guanidinium groups for gene delivery, the polyether‐based copolymers showed particularly efficient transfection at low N/P ratios, along with distinct trends in cytotoxicity linked to polymer composition. Based on the obtained data, further potential improvement of safety and transfection efficiency might be achieved by variation of molar polymer masses and changes of the hydrophobic side chain length. Polycations with lower molar masses are known to exhibit lower cytotoxic effects [103, 104]. For a variation of the length of the charged side chains it is known from the literature that polymers with longer side chains can penetrate better into membranes, which could increase cytotoxicity [102, 111, 112].

In conclusion, these results extend our previous findings and confirm that the guanidinium‐indole motif is transferable across polymer backbones. Overall, these findings identify guanidinium–indole functionalized poly(glycidyl ether)s as promising non‐viral gene delivery systems, with transfection efficiency governed by a finely balanced hydrophilic–hydrophobic ratio, providing a design principle that can be applied to future polymer‐based vectors. These relationships form the basis for fully realizing the potential of these polymers, particularly the best performers, for future in vivo studies. Such studies enable a more intense biological investigation of polyplexes when formed by a therapeutically relevant pDNA and the newly developed polymers regarding biodistribution, immune or inflammation‐relevant side effects, or off‐target gene expression.

## Author Contributions

M.K. and A.D. conceptualization, methodology, investigation, data curation, writing – original draft. L.S. investigation, data curation, writing – review & editing. J.E. and K.C. investigation, data curation, writing – original draft. A.S. investigation, data curation, writing – review & editing. F.H.S. writing – review & editing, supervision, project administration, funding acquisition. D.F. and K.P. conceptualization, writing – review & editing, supervision, project administration, funding acquisition.

## Conflicts of Interest

The authors declare no conflicts of interest.

## Supporting information




**Supporting File**: marc70201‐sup‐0001‐SuppMat.pdf.

## Data Availability

We confirm that the data supporting the findings of our study are fully available within the article and its ESI. No additional data are available outside of these materials.
