# Supplementary material for: Bioinspired, Guanidinium, and Indole Modified Poly(glycidyl ether)s as Highly Efficient Vectors for Polyplex‐Mediated Gene Delivery
Source: Macromol Rapid Commun. 2026 Jan 18;47(6):e00873. doi: 10.1002/marc.202500873 (PMC13003722; doi:10.1002/marc.202500873)
Supplement: Supplementary file 1 — Supporting File: marc70201‐sup‐0001‐SuppMat.pdf. [file MARC-47-e00873-s001.pdf]

Supplementary Information

Bioinspired, Guanidinium and Indole Modified Poly(glycidyl ether)s as Highly Efficient Alternative Vectors for Polyplex-mediated Gene Delivery

Markus Kötzsche[‡ a], Andreas Dzierza[‡ b], Lennert Sölter[a], Jan Egger[b], Kjell Cornelis,[a] Andreas Stihl[a], Felix H. Schacher [a,c], Dagmar Fischer[*b,c,d] and Kalina Peneva[*a,c]

a. Friedrich Schiller University Jena, Institute of Organic and Macromolecular Chemistry (IOMC), Humboldtstr. 10, 07743 Jena, Germany.

b. Friedrich-Alexander-Universität Erlangen-Nürnberg, Division of Pharmaceutical Technology and Biopharmacy, Cauerstr. 4, 91058 Erlangen, Germany.

c. Jena Center for Soft Matter, Philosophenweg 7, 07743 Jena, Germany.

d. FAU NeW - Research Center New Bioactive Compounds, Nikolaus-Fiebiger-Str. 10, 91058 Erlangen, Germany

Chemicals.

All chemicals were purchased from commercial suppliers and used as received unless otherwise specified. Sodium hydroxide (NaOH, 98%, Sigma-Aldrich), epichlorohydrin (ECH, 98%, Sigma-Aldrich), tetrabutylammonium bromide (TBAB, 99%, Sigma-Aldrich), methoxyethanol (99%, Sigma-Aldrich), 3-Indoleethanol (98%, TCI), phosphorus tribromide (PBr_3, 99%, Sigma-Aldrich), potassium thioacetate (98%, Thermo Scientific), N,N′-di-Boc-1H-pyrazole-1-carboxamidine (97%, Carbolution), 1-aminoethanethiol hydrochloride (95%, TCI), tris(2-carboxyethyl)phosphine hydrochloride (TCEP·HCl, 98%, TCI), dry dimethylformamide (99.5%, AcroSeal, FisherScientific), 2-mercaptoethanol (2ME, 99%, Sigma-Aldrich), naphthalene (99%, Sigma Aldrich), potassium (98%, Sigma Aldrich), benzyl alcohol (99%, Sigma Aldrich) and 2,2-dimethoxy-2-phenylacetophenone (DMPAP, 99%, Sigma-Aldrich) were obtained in the stated purities. Allyl glycidyl ether (AGE, 99%, Sigma Aldrich) was vacuum distilled onto activated 4Å molecular sieves and stored in an argon-filled glovebox prior to use in polymerizations.

Monomers and Precursors.

Methoxyethoxy glycidyl ether (MEGE): 96.8 g (2.42 mol, 16 eq) NaOH, 57.8 g (605.4 mmol, 4 eq) epichlorohydrin (ECH), and 2.44 g (7.56 mmol, 0.05 eq) tetrabutylammonium bromide (TBAB) were combined and stirred vigorously at 0 °C. 11.9 mL (11.5 g, 151.3 mmol, 1 eq) methoxyethanol were added dropwise under continuous stirring. After addition, the mixture was allowed to warm to room temperature and stirred overnight. 100 mL deionized water were added, and the mixture was extracted three times with 200 mL diethyl ether. The combined organic phases were dried over anhydrous $MgSO_4$, filtered, and concentrated under reduced pressure to give a yellow oil. The crude product was purified by silica gel column chromatography (ethyl acetate/n-hexane 1/1), followed by vacuum distillation into 4 Å molecular sieves at 90 °C under 10 mbar, yielding 16.7 g (84%) of MEGE as a colorless to pale yellow oil. ^{1}H-NMR (300 MHz, Chloroform-*d*): δ = 3.50 (dd, *J* = 11.6, 3.0 Hz, 1H), 3.37 (m, 2H), 3.26 (m, 2H), 3.12 (m, 1H), 3.09 (s, 3H), 2.85 (m, 1H), 2.48 (dd, *J* = 5.1, 4.1 Hz, 1H), 2.30 (dd, *J* = 5.1, 2.7 Hz, 1H).

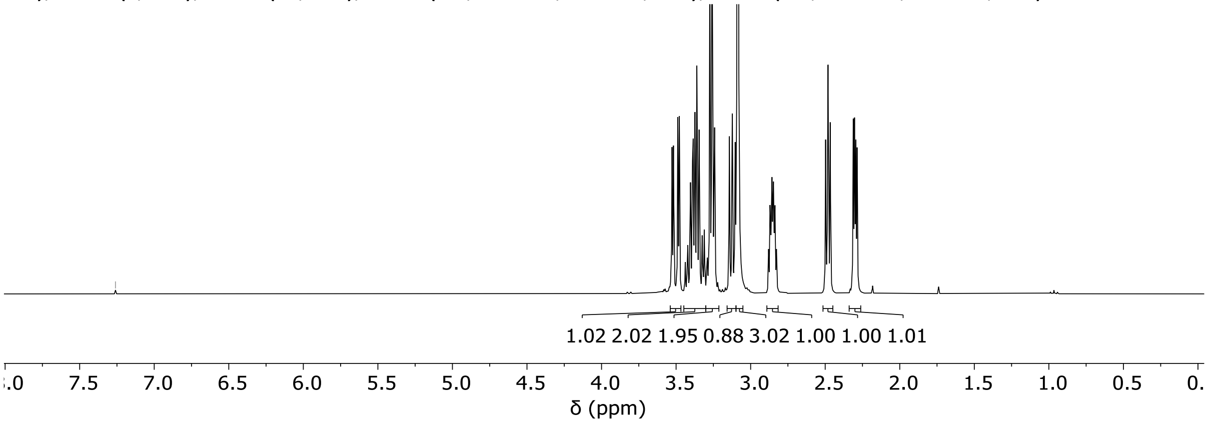

Figure S1. ^{1}H-NMR spectrum of MEGE in $CDCl_3$.

3-(2-bromoethyl)indole: 4.07 g (24.8 mmol, 1 eq) 3-indoleethanol were dissolved in 35 mL ethyl acetate. 1.63 mL (17.4 mmol, 0.7 eq) PBr_3 were added at room temperature. After 3 hours, the excess PBr_3 was quenched with water and the solvent of the organic phase reduced. The product was purified with a silica column (ethyl acetate/n-hexane 2/1). 3.60 g (16.1 mmol, 65%) 3-(2-bromoethyl)indole were obtained. ^{1}H-NMR (300 MHz, Chloroform-*d*): δ = 8.03 (s, 1H), 7.60 (dd, *J* = 7.7, 1.3 Hz, 1H), 7.38 (m, 1H), 7.22 (ddd, *J* = 8.1, 7.0, 1.3 Hz, 1H), 7.15 (ddd, *J* = 8.1, 7.1, 1.2 Hz, 1H), 7.10 (m, 1H), 3.65 (dd, *J* = 8.1, 7.2 Hz, 3H), 3.35 (m, 3H).

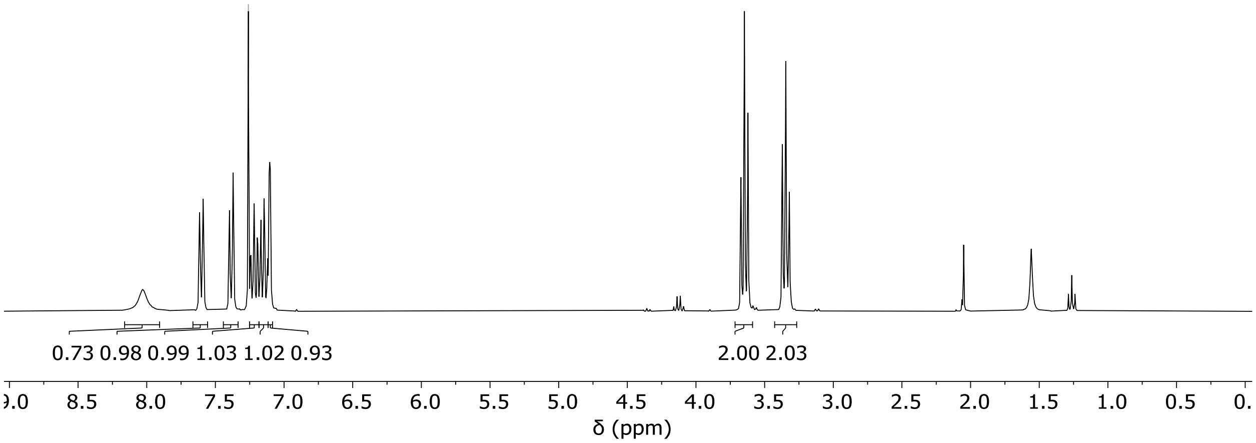

Figure S2. ^{1}H-NMR spectrum of 3-(2-bromoethyl)indole in $CDCl_3$.

S-(2-(1H-indol-3-yl)ethyl) ethanethioate: 3.60 g (16.1 mmol, 1 eq) 3-(2-bromoethyl)indole were dissolved in 50 mL acetone. 2.82 g (24.1 mmol, 1.5 eq) potassiumthioacetate were added and the mixture refluxed for 3 hours. The acetone was removed, the solid dissolved in ethyl acetate, washed with water and concentrated for purification with a silica column (ethyl acetate/n-hexane 1/1). The solvent was removed and 3.60 g (14.9 mmol, 93%, contained 0.25 eq ethyl acetate) S-(2-(1H-indol-3-yl)ethyl) ethanethioate were obtained as brown solid.

^{1}H-NMR (300 MHz, Chloroform-*d*): δ = 7.98 (s, 1H), 7.68 (m, 1H), 7.36 (m, 1H), 7.21 (ddd, *J* = 8.1, 7.0, 1.4 Hz, 1H), 7.14 (ddd, *J* = 8.1, 7.1, 1.2 Hz, 1H), 7.05 (m, 1H), 3.21 (m, 2H), 3.05 (m, 2H), 2.35 (s, 3H).

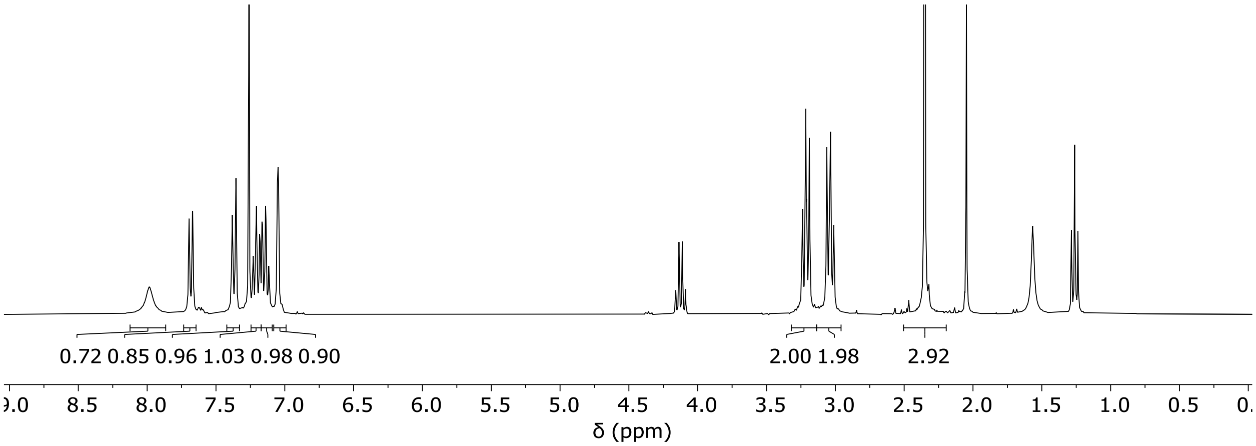

Figure S3. ^{1}H-NMR spectrum of 3 S-(2-(1H-indol-3-yl)ethyl) ethanethioate in $CDCl_3$.

2-(1H-indol-3-yl)ethane-1-thiol (IET): 3.60 g (14.9 mmol, 1 eq, contained 0.25 eq ethyl acetate) S-(2-(1H-indol-3-yl)ethyl) ethanethioate were dissolved in 120 mL methanol. 0.80 g (20.0 mmol, 1.4 eq) sodium hydroxid were added and the mixture refluxed for 2 hours. Then, the mixture was neutralized with concentrated HCl in water. The product was extracted with dichloromethane, washed with water and the solvent of the organic phase removed. 2.73 g (14.9 mmol, quantitative) 2-(1H-indol-3-yl)ethane-1-thiol were obtained as mixture of the free thiol and the disulfide.

^{1}H-NMR (300 MHz, Chloroform-*d*): δ = 7.95 (s, 1H), 7.60 (d, *J* = 7.8 Hz, 1H), 7.36 (m, 1H), 7.21 (m, 1H), 7.12 (m, 1H), 7.02 (m, 1H), 3.17 (m, 2H), 3.06 (m, 2H), 2.88 (q, *J* = 7.4 Hz, 2H), 1.46 (t, *J* = 7.8 Hz, 1H).

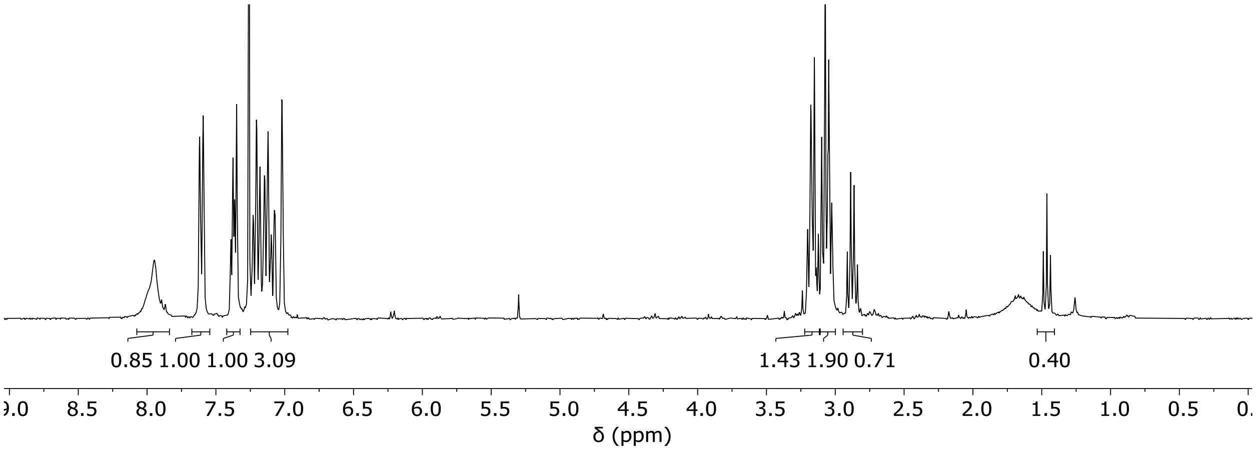

Figure S4. ^{1}H-NMR spectrum of 2-(1H-indol-3-yl)ethane-1-thiol in $CDCl_3$.

N,N'-di-Boc-N''-(2-mercaptoethyl)guanidine: 10.0 g (32.2 mmol, 1 eq) N,N'-di-Boc-1H-pyrazole-1-carboxamidine were dissolved in 50 mL acetonitrile. 7.33 g (64.4 mmol, 2 eq) 1-aminoethanethiol hydrochloride dissolved in 50 mL acetonitrile, 15 mL water and 45 mL (322 mmol, 10 eq) triethylamine were added. Within 24 hours, the product precipitated and the suspension was diluted with 400 mL water, filtered, washed with water and dried. 8.0 g (24.0 mmol, 74.5%) N,N'-di-Boc-N''-(2-mercaptoethyl)guanidine were obtained as white solid and a mixture of the free thiol and the disulfide. ^{1}H-NMR (300 MHz, Chloroform-*d*): δ = 11.47 (s, 1H), 8.67 (m, 1H), 3.78 (q, *J* = 6.1 Hz, 2H), 3.65 (q, *J* = 6.2 Hz, 2H), 2.87 (t, *J* = 6.3 Hz, 2H), 2.73 (dt, *J* = 8.6, 6.5 Hz, 2H), 1.49 (s, 18H).

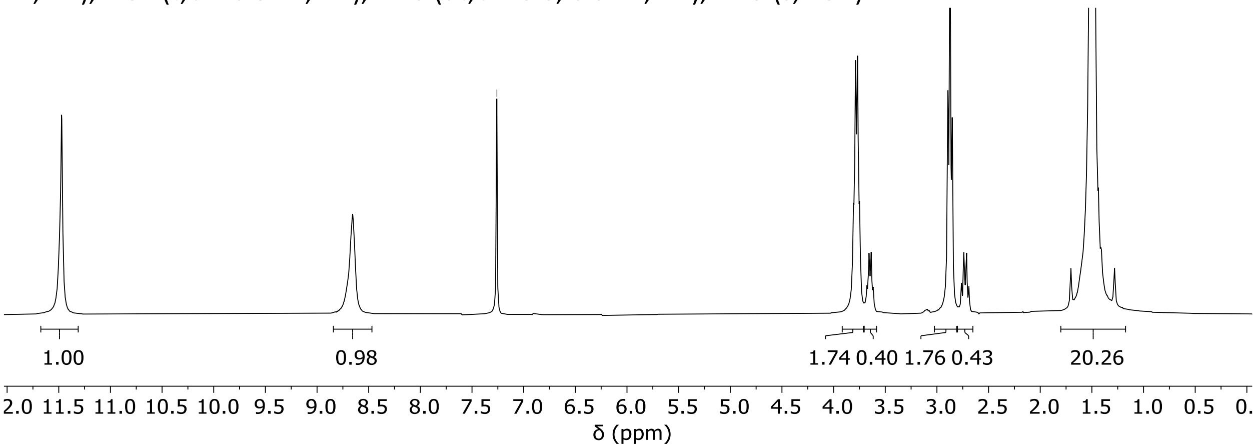

Figure S5. ^{1}H-NMR spectrum of N,N'-di-Boc-N''-(2-mercaptoethyl)guanidine in $CDCl_3$.

Deprotection, (2-mercaptoethyl)guanidine (MEG): 8.0 g (24.0 mmol, 1 eq) N,N'-di-Boc-N''-(2-mercaptoethyl)guanidine were dissolved in 70 mL dichloromethane and 25 mL (326 mmol, 14 eq) trifluoroacetic acid. After 24 hours, the solvent was removed and (2-mercaptoethyl)guanidine obtained as trifluoroacetate salt (4.83 g, 0.6 eq trifluoroacetic acid) and a mixture of the free thiol and the disulfide. ^{1}H-NMR (300 MHz, Deuterium Oxide): δ = 3.54 (t, *J* = 6.2 Hz, 1H), 3.39 (q, *J* = 6.5 Hz, 2H), 2.91 (dd, *J* = 6.7, 5.8 Hz, 2H), 2.82 (t, *J* = 6.6 Hz, 2H), 2.72 (t, *J* = 6.5 Hz, 2H).

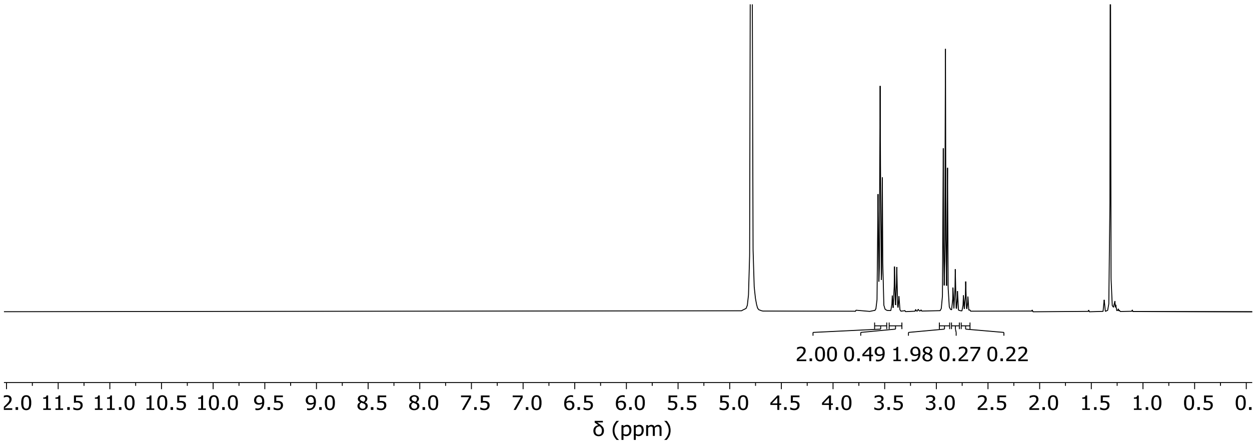

Figure S6. ^{1}H-NMR spectrum of (2-mercaptoethyl)guanidine in D_2O.

Unfunctionalized poly(glycidyl ether)

The polymerization reactions were performed as described with the amounts listed in Table S1.

Table S1. Feed composition for the polymerization of oxirane monomers. Listed are the amounts of allyl glycidyl ether (AGE) and 2-methoxyethyl glycidyl ether (MEGE) used in the respective reactions.

Polymer	AGE	MEGE	Initiator solution
**P(AGE-*stat*-MEGE)**	602 mg	177 mg	0.45 mL
	5.27 mmol	1.34 mmol	0.066 mmol
	80 eq	20 eq	1 eq
**P(AGE)**	801 mg		0.48 mL
	7.02 mmol	-	0.070 mmol
	100 eq		1 eq

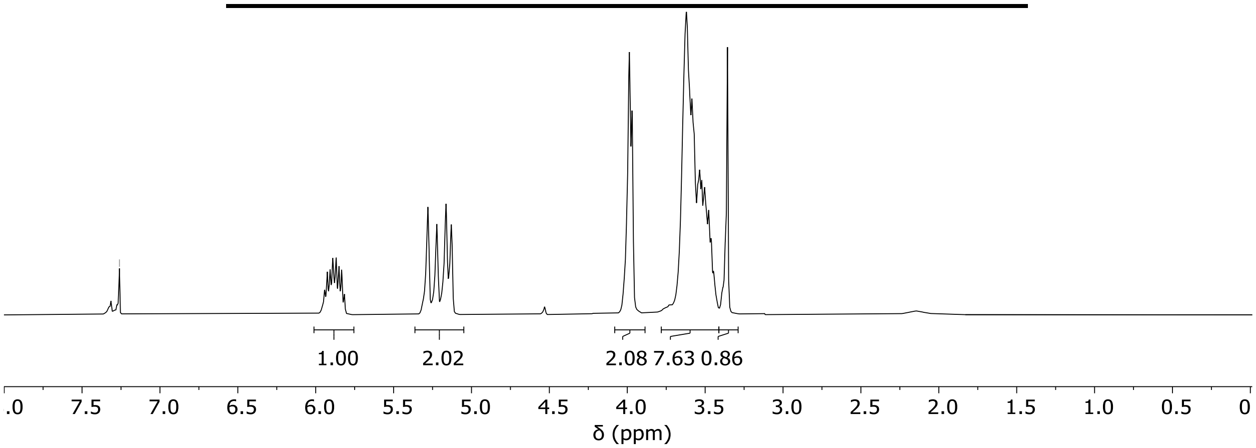

Figure S7. ^{1}H-NMR spectrum of P(AGE-*stat*-MEGE) in $CDCl_3$.

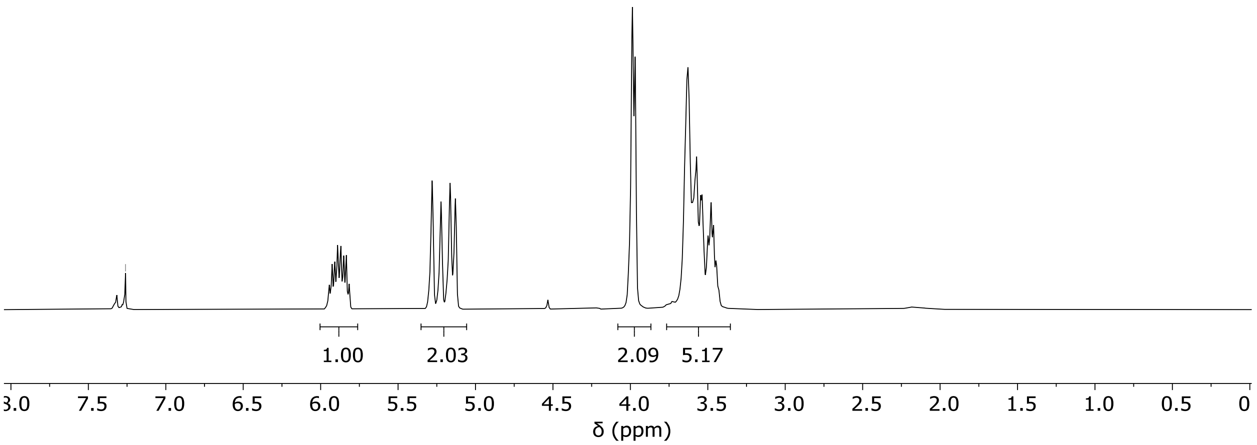

Figure S8. ^{1}H-NMR spectrum of P(AGE) in $CDCl_3$.

Size Exclusion Chromatography was performed in THF as the eluent at 40 °C using PSS SDV 5 µm VS/100/1000/100000 Å columns at a flow rate of 1 mL/min. A refractive indes detector was used and the calibration was done with PEG standards.

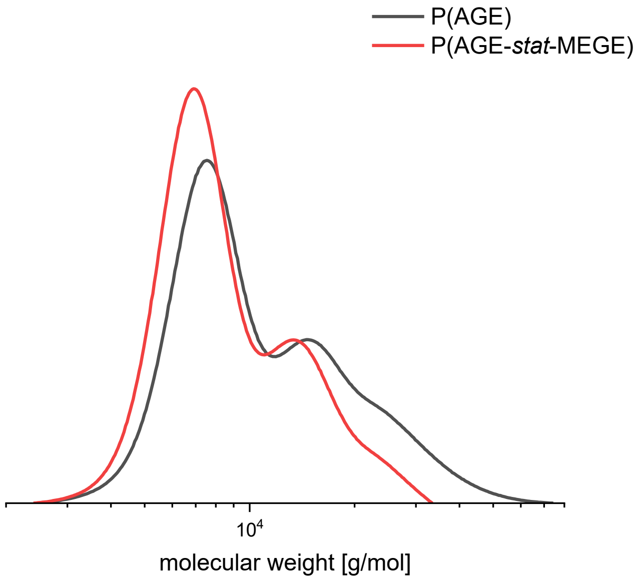

Figure S9. SEC curves of the unfunctionalised poly(glycidyl ether)s in THF with PEG calibration.

Grafted poly(glycidyl ether)
The copolymers were grafted as described with the amounts listed in Table S2.

Table S2. Used amount for the first grafting of the poly(glycidyl ether)s with (2-mercaptoethyl)guanidine (MEG) and 2-(1H-indol-3-yl)ethane-1-thiol (IET).

**Polymer**	**P(AGE)**	**MEG**	**IET**	**TCEP HCl**	**DMPAP**	**DMF**
**$P(G_{92}H_8)$**	100 mg<br>876 µmol<br>1 eq	388 mg<br>1.93 mmol<br>2.2 eq	-	477 mg<br>1.66 mmol<br>1.9 eq	27 mg<br>104 µmol<br>0.12 eq	1.5 mL
**$P(G_{82}I_7H_{11})$**	100 mg<br>876 µmol<br>1 eq	369 mg<br>1.84 mmol<br>2.1 eq	15 mg<br>87 µmol<br>0.1 eq	477 mg<br>1.66 mmol<br>1.9 eq	27 mg<br>104 µmol<br>0.12 eq	1.5 mL
**$P(G_{72}I_{11}H_{17})$**	100 mg<br>876 µmol<br>1 eq	349 mg<br>1.74 mmol<br>2.0 eq	29 mg<br>166 µmol<br>0.2 eq	477 mg<br>1.66 mmol<br>1.9 eq	27 mg<br>104 µmol<br>0.12 eq	1.5 mL
**$P(G_{65}I_{22}H_{13})$**	100 mg<br>876 µmol<br>1 eq	310 mg<br>1.54 mmol<br>1.76 eq	59 mg<br>333 µmol<br>0.4 eq	477 mg<br>1.66 mmol<br>1.9 eq	27 mg<br>104 µmol<br>0.12 eq	1.5 mL
	**P(AGE-*stat*-MEGE)**	**MEG**	**IET**	**TCEP HCl**	**DMPAP**	**DMF**
**$P(G_{77}M_{22}H_1)$**	100 mg<br>650 µmol<br>1 eq	362 mg<br>1.80 mmol<br>2.8 eq	-	445 mg<br>1.55 mmol<br>2.4 eq	25 mg<br>98 µmol<br>0.15 eq	1.5 mL
**$P(G_{69}M_{22}I_5H_4)$**	100 mg<br>650 µmol<br>1 eq	344 mg<br>1.71 mmol<br>2.6 eq	14 mg<br>79 µmol<br>0.12 eq	445 mg<br>1.55 mmol<br>2.4 eq	25 mg<br>98 µmol<br>0.15 eq	1.5 mL
**$P(G_{64}M_{22}I_{10}H_4)$**	100 mg<br>650 µmol<br>1 eq	326 mg<br>1.62 mmol<br>2.5 eq	28 mg<br>158 µmol<br>0.24 eq	445 mg<br>1.55 mmol<br>2.4 eq	25 mg<br>98 µmol<br>0.15 eq	1.5 mL
**$P(G_{50}M_{22}I_{20}H_8)$**	100 mg<br>650 µmol<br>1 eq	290 mg<br>1.44 mmol<br>2.2 eq	55 mg<br>310 µmol<br>0.48 eq	445 mg<br>1.55 mmol<br>2.4 eq	25 mg<br>98 µmol<br>0.15 eq	1.5 mL

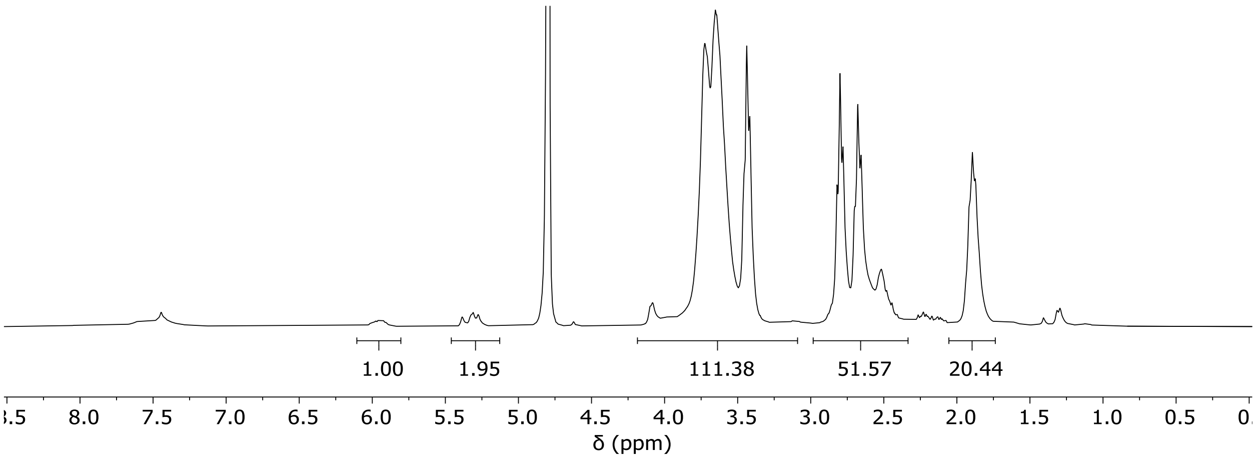

Figure S10. ^{1}H-NMR spectrum of the polymer $P(G_{92}A_8)$ in D_2O.

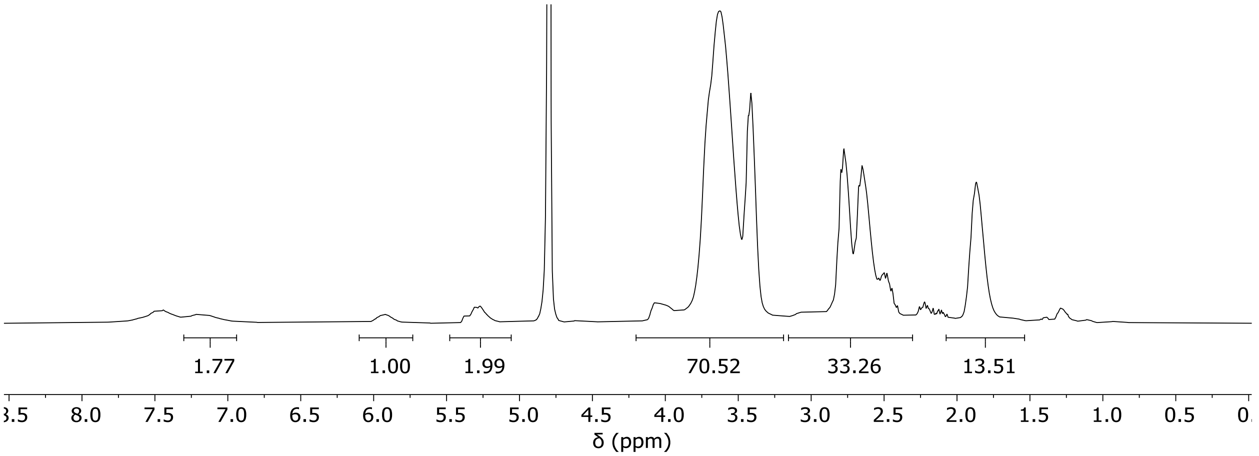

Figure S11. ^{1}H-NMR spectrum of the polymer $P(G_{82}I_7A_{11})$ in D_2O.

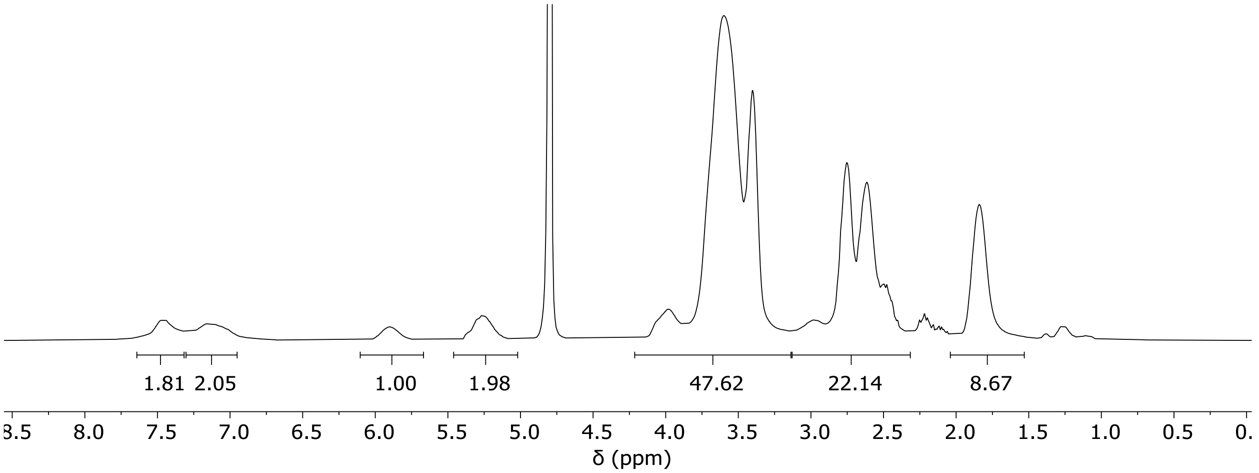

Figure S12. ^{1}H-NMR spectrum of the polymer $P(G_{72}I_{11}A_{17})$ in D_2O.

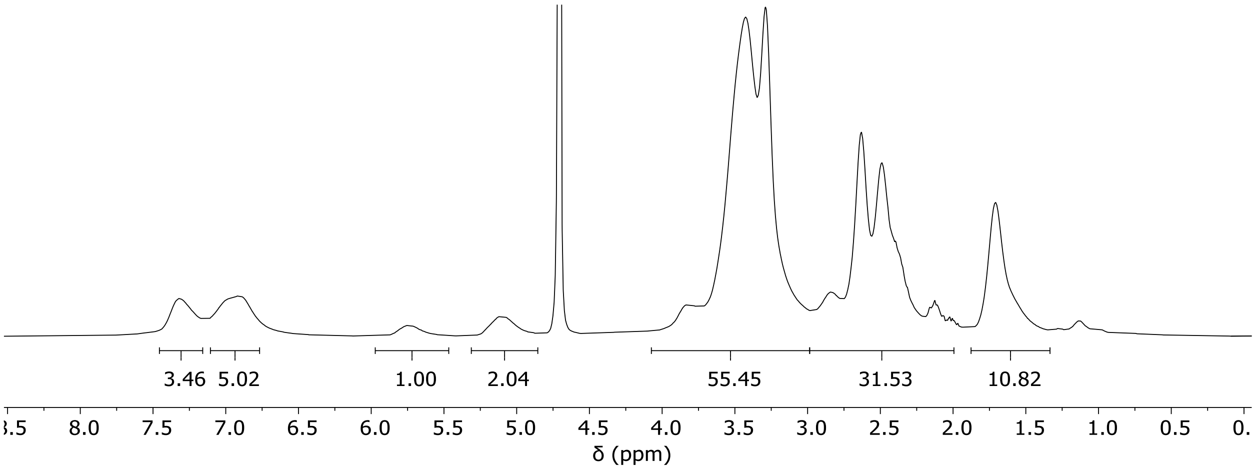

Figure S13. ^{1}H-NMR spectrum of the polymer $P(G_{65}I_{22}A_{13})$ in D_2O.

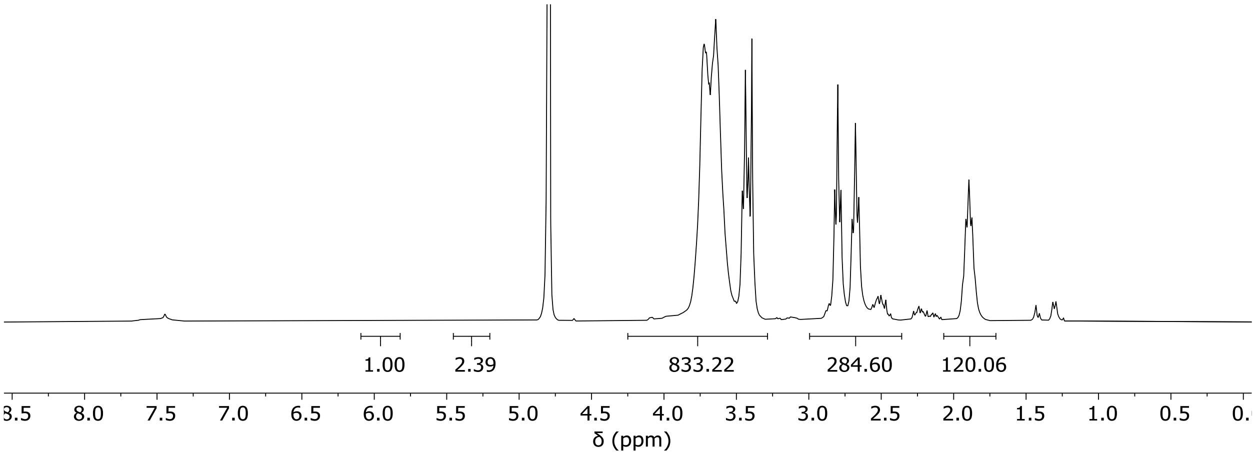

Figure S14. ^{1}H-NMR spectrum of the polymer $P(G_{77}M_{22}A_{1})$ in D_2O.

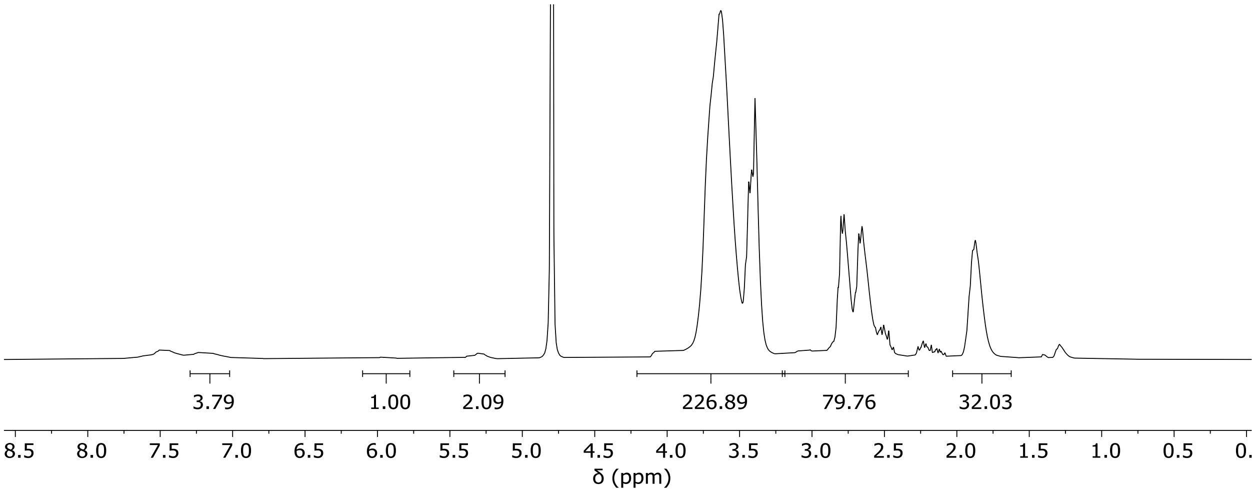

Figure S15. ^{1}H-NMR spectrum of the polymer $P(G_{69}M_{22}I_{5}A_{4})$ in D_2O.

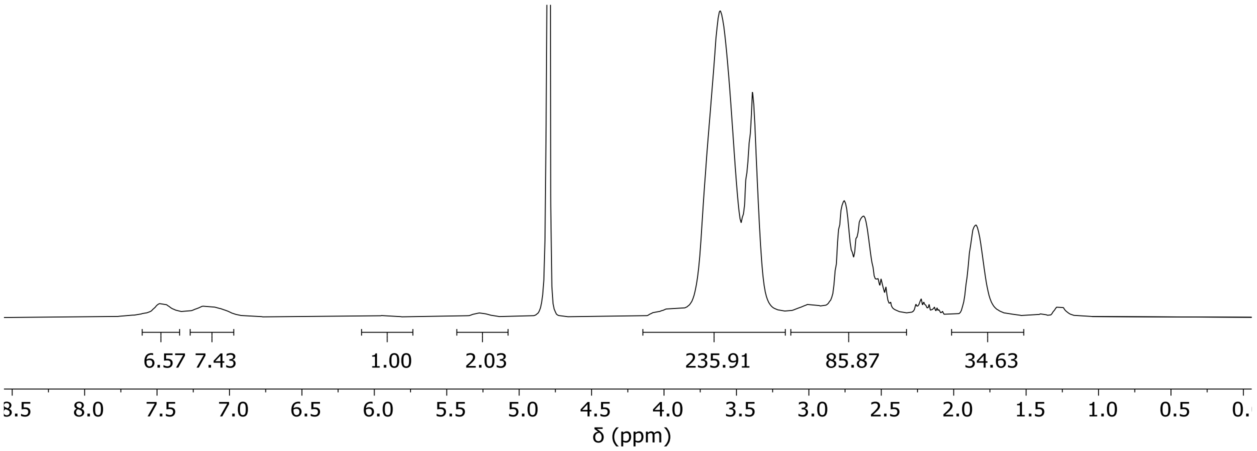

Figure S16. ^{1}H-NMR spectrum of the polymer $P(G_{64}M_{22}I_{10}A_{4})$ in D_2O.

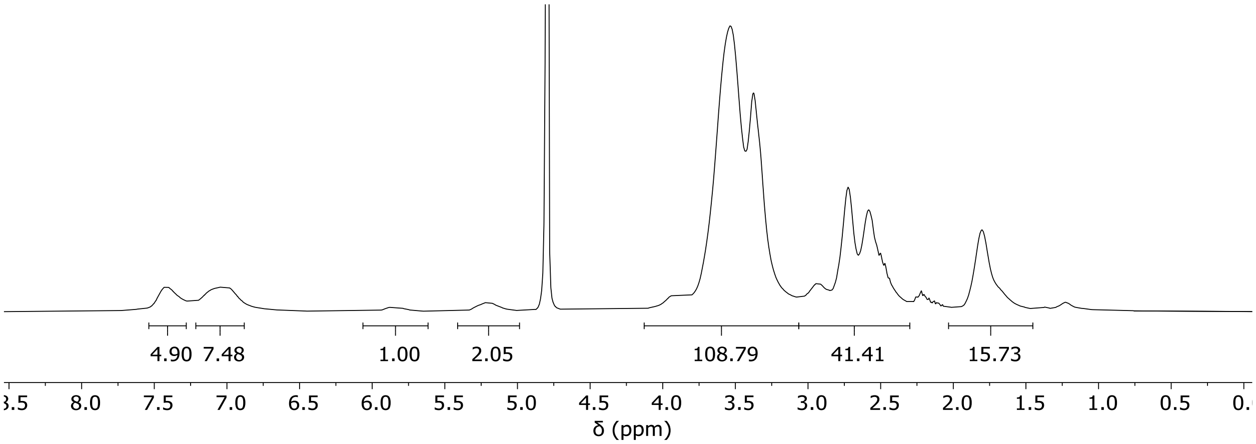

Figure S17. ^{1}H-NMR spectrum of the polymer $P(G_{50}M_{22}I_{20}A_8)$ in D_2O.

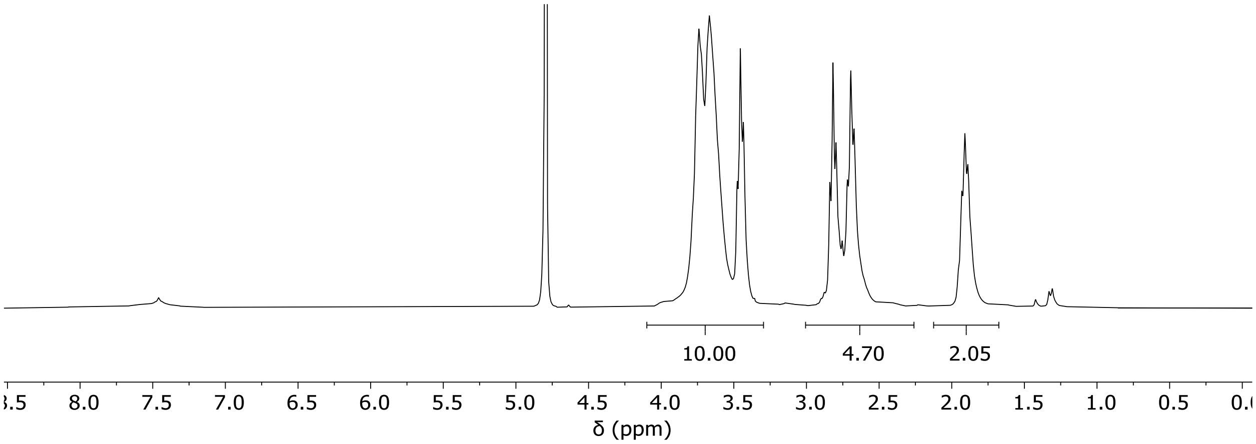

Figure S18. ^{1}H-NMR spectrum of the polymer $P(G_{92}H_8)$ in D_2O.

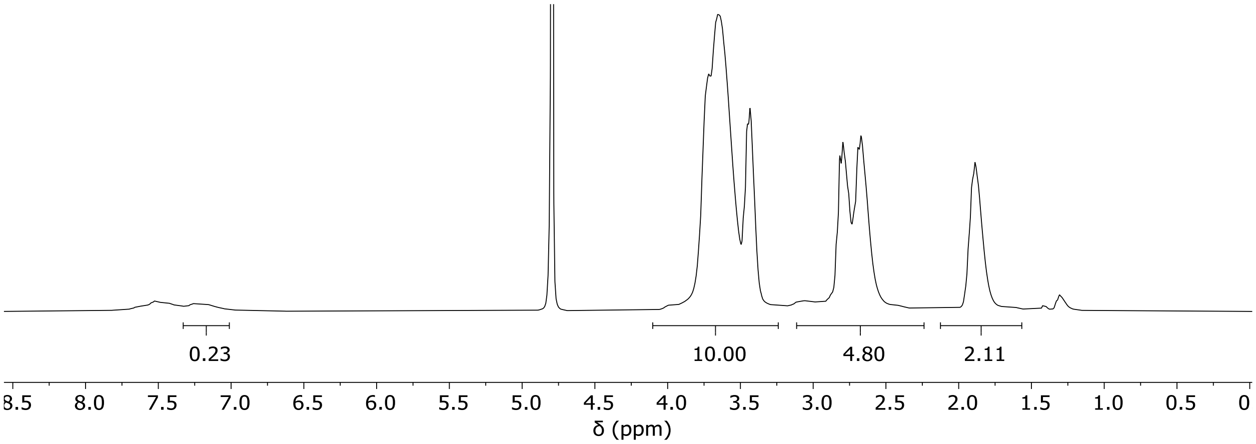

Figure S19. ^{1}H-NMR spectrum of the polymer $P(G_{82}I_7H_{11})$ in D_2O.

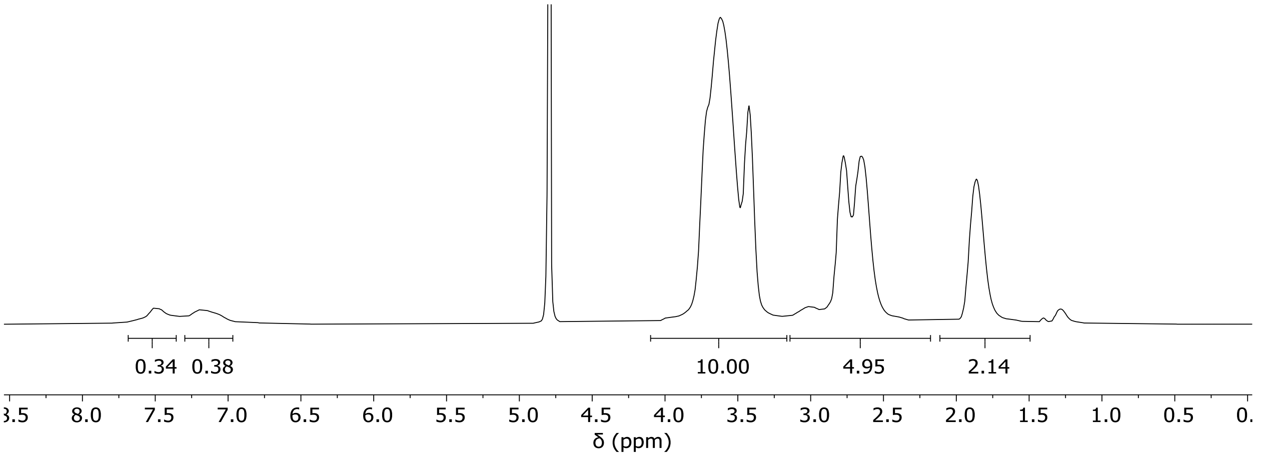

Figure S20. ^{1}H-NMR spectrum of the polymer P($G_{72}I_{11}H_{17}$) in D_2O.

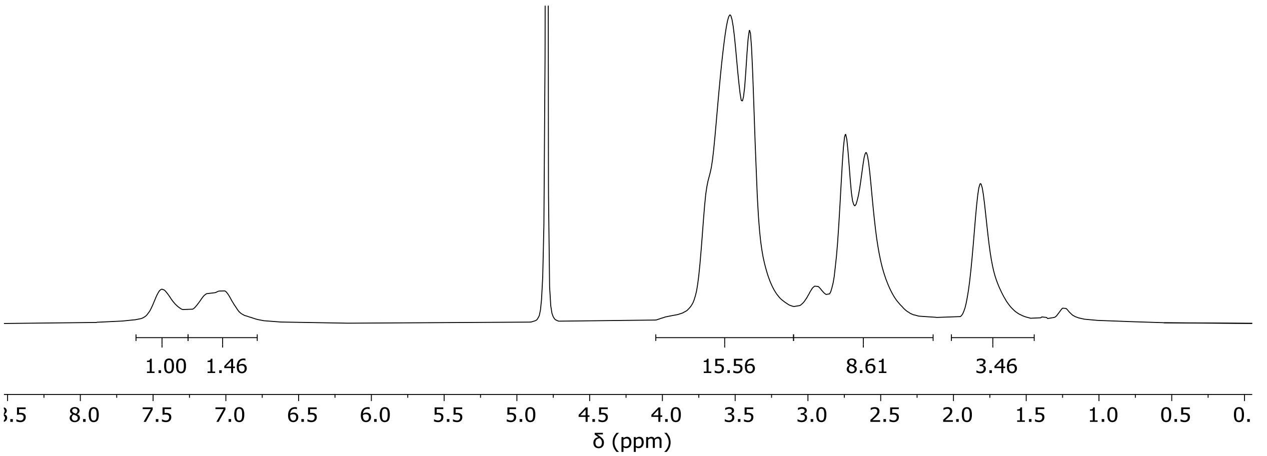

Figure S21. ^{1}H-NMR spectrum of the polymer P($G_{65}I_{22}H_{13}$) in D_2O.

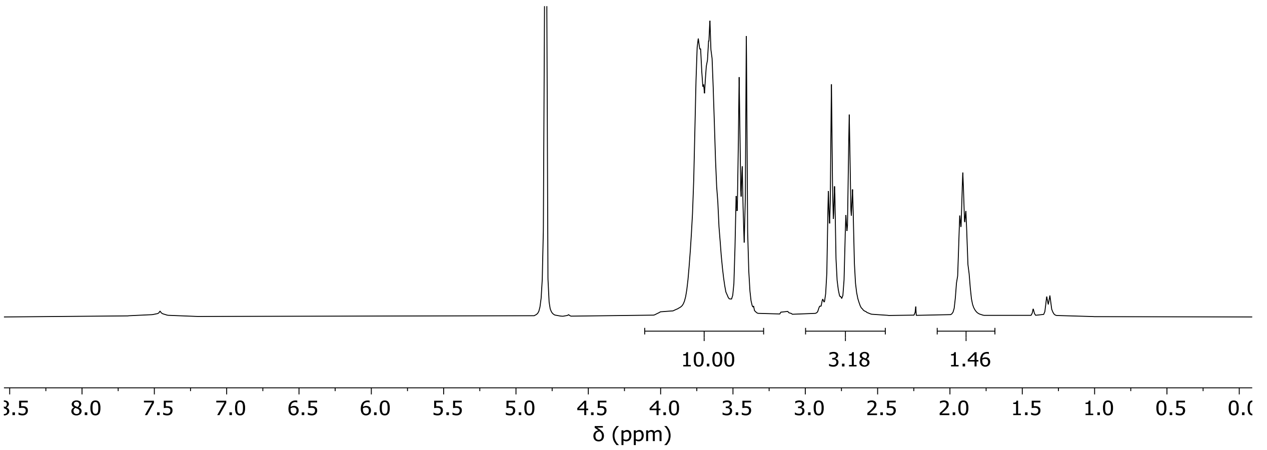

Figure S22. ^{1}H-NMR spectrum of the polymer P($G_{77}M_{22}H_1$) in D_2O.

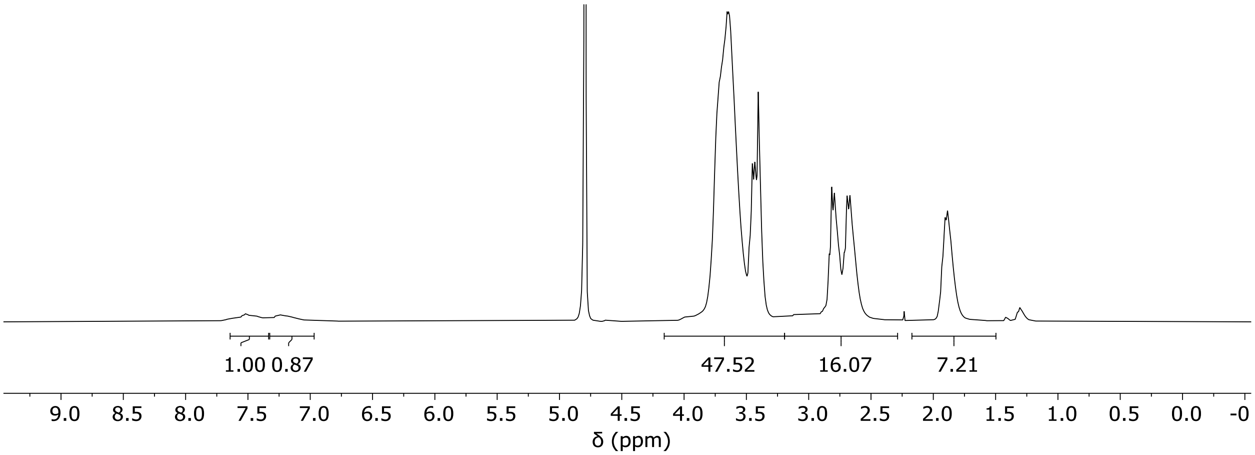

Figure S23. ^{1}H-NMR spectrum of the polymer $P(G_{69}M_{22}I_{5}H_{4})$ in D_2O.

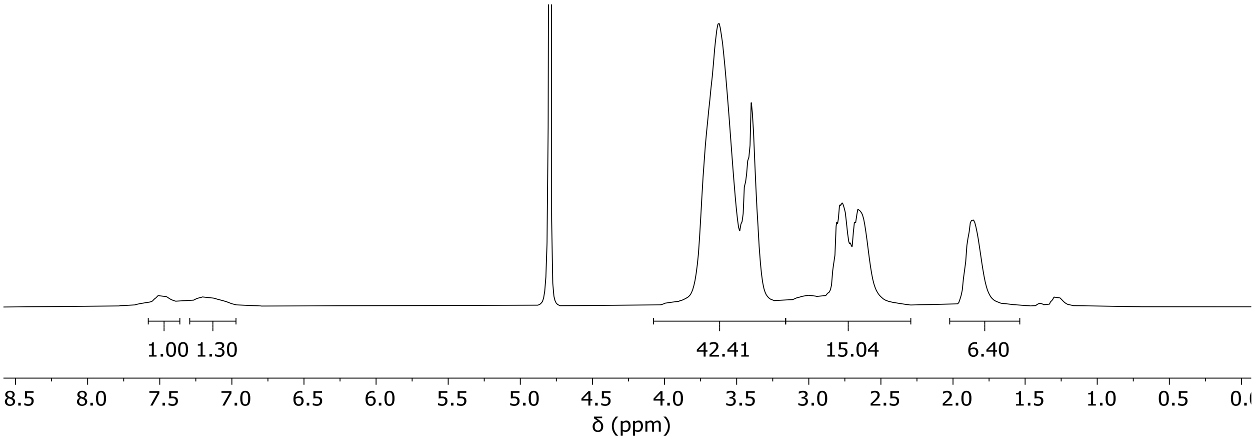

Figure S24. ^{1}H-NMR spectrum of the polymer $P(G_{64}M_{22}I_{10}H_{4})$ in D_2O.

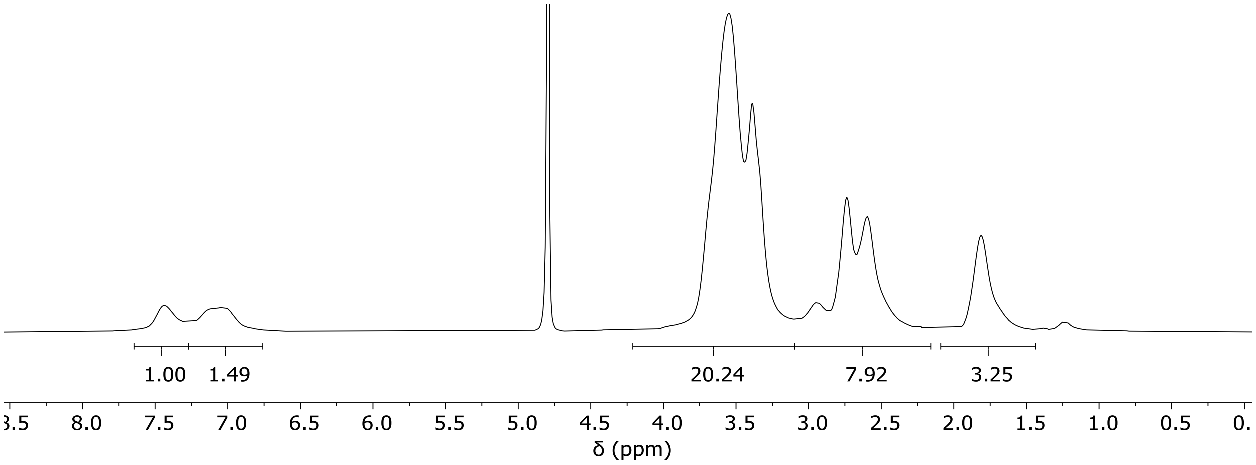

Figure S25. ^{1}H-NMR spectrum of the polymer $P(G_{50}M_{22}I_{20}H_{8})$ in D_2O.

Size exclusion chromatography was performed in dimethylacetamide and 0.21% lithium chloride as the eluent at 40 °C using PSS GRAM guard/30/1000 Å columns at a flow rate of 1 mL/min. A G1362A refractive index detector and a G1315D UV detector (290 nm) were used and the calibration was done with poly(ethylene glycol) (PEG) standards.

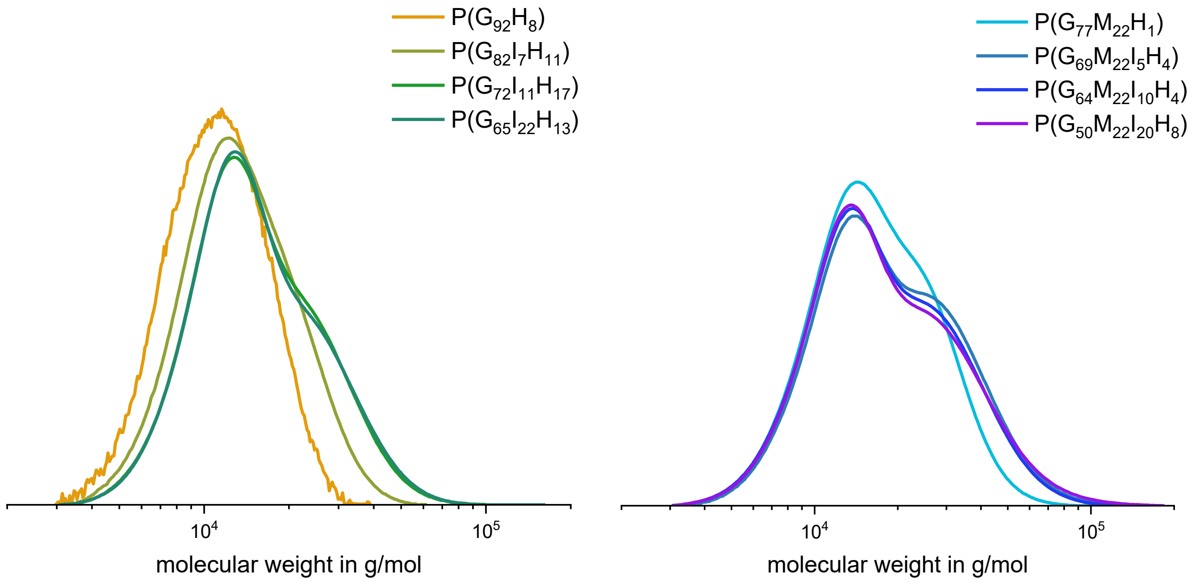

Figure S26. SEC curves of the functionalized poly(glycidyl ether)s in dimethylacetamide with PEG calibration.

We attribute the shoulder at approximately double molar mass to chain-chain coupling occurring during the polymerization, presumably due to side reactions of the AGE units at high conversion.

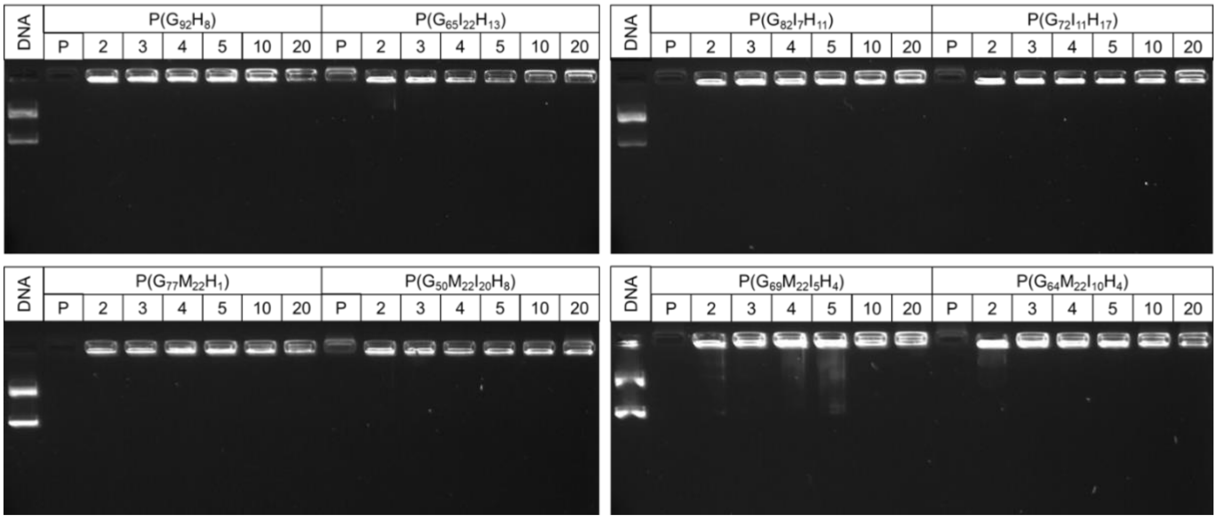

Figure S27. Agarose gel electrophoresis with polyplexes at different N/P ratios (2-20), using GelRed® as fluorescent intercalating dye. Free plasmid DNA (D) and free respective polymer as in N/P 20 (P) without pDNA were used as controls.

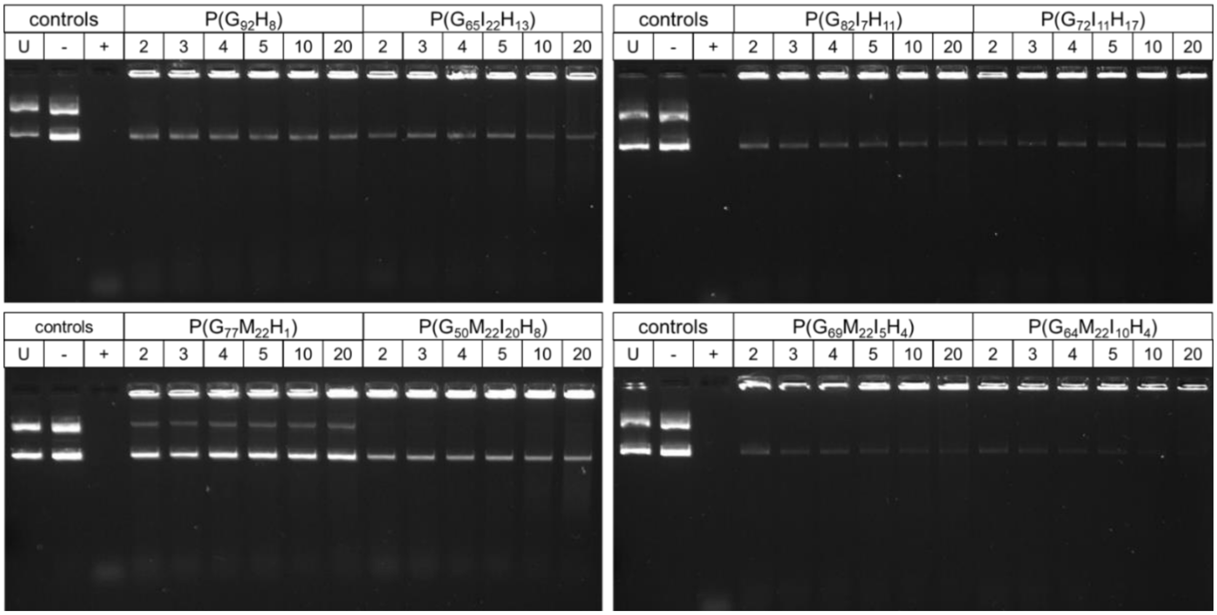

Figure S28. Protection of pDNA from degradation through DNase I. Polyplexes were incubated with DNase I and pDNA was displaced from the polyplexes with heparin after inactivation of the enzyme. Untreated pDNA (U), pDNA undergoing the same thermal stress as the polyplexes, but without adding DNase (-) and pDNA treated the same way as the polyplexes (+) were used as controls.

Table S3. Calculated IC_{50}-values of the polymers. Calculations were performed using logistic function in Origin® 2021. Numbers in brackets represent the standard error of the calculated values given by the program.

Polymer	IC_{50} [µg /mL]	Polymer	IC_{50} [µg /mL]
**$P(G_{92}H_8)$**	16.0 (1.4)	**$P(G_{77}M_{22}H_1)$**	10.8 (1.0)
**$P(G_{82}H_{11}I_7)$**	13.3 (0.7)	**$P(G_{69}M_{22}H_4I_5)$**	8.5 (0.5)
**$P(G_{72}H_{17}I_{11})$**	15.7 (1.8)	**$P(G_{64}M_{22}H_4I_{10})$**	8.8 (0.4)
**$P(G_{65}H_{13}I_{22})$**	16.8 (1.0)	**$P(G_{50}M_{22}H_8I_{20})$**	11.5 (0.8)
